# Personalized Cancer Immunotherapy Boosted by cGAS‐STING‐Targeted Nanovaccines in Combination With Nutrient Modulation

**DOI:** 10.1002/EXP.20240183

**Published:** 2025-08-22

**Authors:** Wenping Huang, Guoliang Cao, Mixiao Tan, Fuhao Jia, Jie Zhang, Wen Su, Yue Yin, Hai Wang

**Affiliations:** ^1^ CAS Key Laboratory for Biomedical Effects of Nanomaterials & Nanosafety CAS Center for Excellence in Nanoscience National Center for Nanoscience and Technology Beijing China; ^2^ State Key Laboratory of Clean and Efficient Turbomachinery Power Equipment Department of Mechanical Engineering Tsinghua University Beijing China; ^3^ University of Chinese Academy of Sciences Beijing China; ^4^ Department of Pharmaceutical Sciences University of Michigan Ann Arbor Michigan USA; ^5^ School of Medical Technology Beijing Institute of Technology Beijing China; ^6^ Advanced Technology Research Institute Beijing Institute of Technology Jinan China

**Keywords:** personalized cancer immunotherapy, STING, 2'3'‐cGAMP, nanovaccine, fasting‐mimicking diet

## Abstract

Cyclic dinucleotides, which act as agonists for the stimulator of interferon genes (STING), are pivotal in stimulating both adaptive and innate immune reactions for advancing cancer immunotherapy. However, their therapeutic potential is hampered by inherent limitations, including susceptible degradation and inefficient delivery. Herein, we design genetically engineered bacteria (2'3'‐cGAMP@*E.coli*) capable of producing 2'3'‐cGAMP in the cytoplasm and then fabricate personalized nanovaccines (nECTs) by assembling 2'3'‐cGAMP@*E.coli* with autologous tumor antigens instead of complicated chemical synthesis. Our in vitro analysis confirms that nECTs are capable of potently stimulating dendritic cell activity and amplifying the cross‐presentation of antigens by leveraging the STING signaling route, underscoring their potential to bolster immune response priming. Translating these findings into in vivo models, vaccination with nECTs leads to a pronounced infiltration of effector T cells into tumor sites, concurrent with an IFN‐β‐mediated remodeling of the suppressive tumor microenvironment by innate immune cells. Notably, the therapeutic efficacy of nECTs is further augmented when coupled with a fasting‐mimicking diet regimen, highlighting the synergistic potential of this combinatory strategy. Collectively, this dual modality represents a significant stride towards enhancing the precision and effectiveness of immunotherapeutic interventions in oncology.

## Graphical abstract

The engineered bacteria (2'3'‐cGAMP@*E.coli*) is designed to synthesize intracellular 2'3'‐cGAMP, enabling the assembly of personalized nanovaccines (nECTs) through a bioconjugation with autologous tumor antigens, circumventing elaborate chemical production processes. The therapeutic impact of nECTs is significantly amplified when combined with a fasting‐mimicking diet, illustrating the potent synergies of this integrated therapeutic approach.

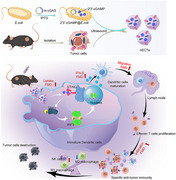

## 1 Introduction

Cancer immunotherapy has been extensively studied for treating many cancer types and has yielded encouraging therapeutic results [1, 2, 3]. Therapeutic cancer vaccines, a key aspect of active immunotherapy, have garnered significant interest [4, 5]. The autologous tumor cell vaccine is a promising approach to providing diverse tumor antigens [6, 7]. Nonetheless, numerous resistance mechanisms to immunotherapy are documented, including an immunosuppressive tumor microenvironment that impedes response to checkpoint inhibitors in certain patients, thereby reducing therapeutic efficacy [8, 9]. Meanwhile, tumor immunosuppressive factors can directly suppress innate immune and effector T cells, significantly limiting their effective anti‐tumor response [10, 11]. In addition, immune checkpoint inhibitors can enhance the ability of an immune system to combat tumors by alleviating the inhibition of T cells. However, this approach can also result in systemic immune activation, thereby increasing the risk of autoimmune disorders and other adverse inflammatory responses [12, 13]. Therefore, promoting immune stimulation in favor of tumor‐infiltrating lymphocytes (TILs) and remodeling innate immune cells within the tumor immunosuppressive milieu has emerged as a pivotal strategy in antitumor immunotherapy.

Activating the stimulator of interferon genes (STING) pathway as a means to stimulate both adaptive and innate immune responses represents an attractive strategy for boosting the immunogenicity of tumors and generating robust antitumor immunity [14, 15, 16]. STING, located in the endoplasmic reticulum, facilitates the identification of endogenous and exogenous nucleic acid ligands without directly detecting cytoplasmic DNA [17, 18]. Cyclic GMP‐AMP synthetase (cGAS) serves as the sensor for DNA within the cytoplasm, triggering the synthesis of cGAMP [19, 20]. This molecule then proceeds to activate the STING protein and resulting in the generation of type I interferons (IFN‐I) and various proinflammatory cytokines [21, 22, 23]. Of these, IFN‐I and pro‐inflammatory factors are essential for the development and functionality of antigen‐presenting cells (APCs), with a significant impact on dendritic cells (DCs) function. These components are indispensable for the activation of T cells specific to tumor antigens [24, 25], and activation or reprogramming of innate immune components, such as natural killer cells and macrophages, significantly amplifies the body's anti‐tumor defense mechanisms [26, 27]. Several STING agonists have been investigated to achieve exogenous STING activation, demonstrating promising antitumor therapeutic outcomes in preclinical models [28, 29]. Nevertheless, inefficient drug delivery, rapid clearance due to metabolism, and systemic toxicity limit their activity and therapeutic efficacy [30, 31].

**FIGURE 1:**
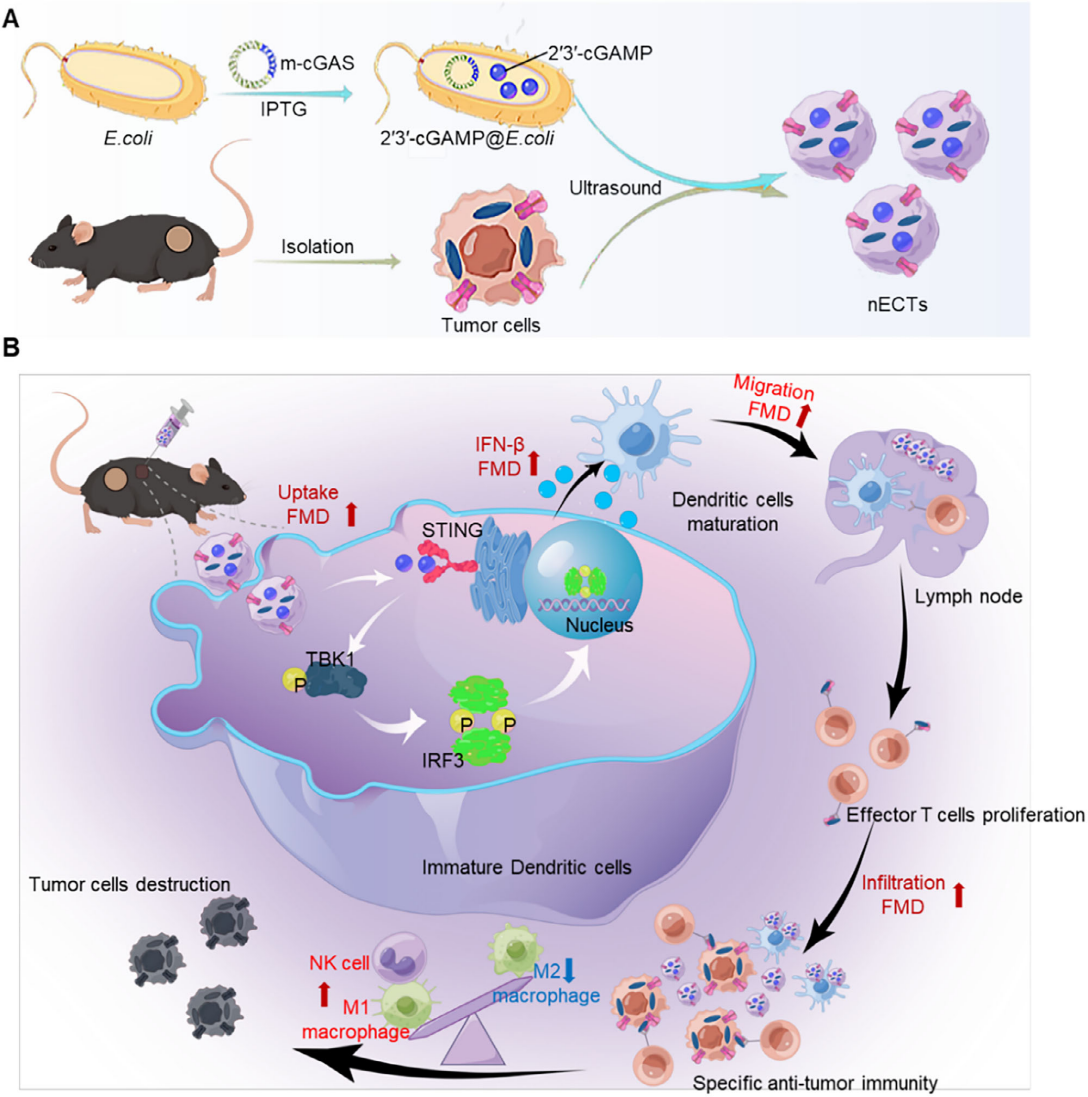
Schematic illustration of nECTs‐mediated 2'3'‐cGAMP‐driven STING activation for enhancing cancer immunotherapy. (A) Tumor cells and 2'3'‐cGAMP@*E.coli* were fabricated to form nECTs through ultrasound sonication. The nECTs could deliver both 2'3'‐cGAMP and whole tumor antigens in APCs for triggering effective STING activation and antigen cross‐presentation. (B) The proposed mechanism of nECTs‐mediated 2'3'‐cGAMP could induce STING activation. In particular, FMD treatment could enhance the cellular uptake of nECTs and promote ER stress and LC3 lipidation to trigger STING translocation from the endoplasmic reticulum to the Golgi. Then, the expressions of p‐TBK1 and p‐IRF3 were upregulated for IFN‐β secretion, thereby triggering both innate immunity and adaptive immunity for enhancing cancer immunotherapy. The schematic diagram was drawn using FigDraw.

Nanotechnology can improve pharmacokinetic profiles and facilitate the cytoplasmic delivery of STING agonists [32, 33]. Recent advances in nanotechnology have shown promise in overcoming existing challenges. PEGylated liposomes enhance STING agonist stability, prolong circulation, and boost cellular uptake, optimizing antitumor immunity with reduced side effects [34]. Cationic lipid nanoparticles improve intracellular release by stabilizing lysosomal pH and preventing antigen degradation [35, 36]. Engineered extracellular vesicles, like exosomes, offer enhanced selectivity and efficacy owing to their cell‐specific origins, significantly boosting intracellular delivery and tumor suppression of STING agonists [37, 38]. Nevertheless, most of these nanocarriers involve the loading process of STING agonists and aim to improve targeting efficiency at the tumor site. In contrast, our study utilizes nano protoplasts as the primary production sites for STING agonists, thereby eliminating the need for additional loading processes and enabling the simultaneous integration of multiple tumor antigens for efficient delivery into APCs. Specifically, we fabricated genetically engineered bacteria (2'3'‐cGAMP@*E.coli*) by heterologous expression of a cluster of genes encoding mouse‐cGAS (m‐cGAS) that can be heterologously expressed using plasmids. Then, cGAS is activated by binding to cytosolic DNA, leading to the production of cGAMP. After synthesis, cGAMP typically localizes in the cytoplasm of bacteria [39]. We then assembled autologous tumor cells to prepare personalized nanovaccines (nECTs) (Figure 1A). Thus, nECTs could efficiently deliver 2′3′‐cGAMP alongside self‐tumor antigens, facilitating the maturation of DCs and enhancing the presentation of antigens. This technology platform allows the production of 2′3′‐cGAMP from cheaper materials, providing a sustainable source of cyclic dinucleotides for scientific research and clinical translation. Particularly, nECTs could initiate adaptive immune effects in subcutaneous sites, draining LNs, and entering tumor tissues to trigger IFN‐β‐driven innate immune effects. In addition, the fasting‐mimicking diet (FMD) [40, 41] was utilized for boosting the cellular absorption of nECTs within APCs, further upregulating the activation of STING signaling and IFN‐β secretion in DCs. Consequently, nECTs efficiently induced infiltration of antigen‐specific T cells into tumor tissues, remodeled the tumor immunosuppressive microenvironment, and inhibited the growth of CT26 colon, 4T1 breast, B16‐F10, or B16‐OVA melanoma tumors (Figure 1B). Overall, this study demonstrates the potential of nECTs as a platform to stimulate STING and thereby augment cancer immunotherapy effectively.

## 2 Results and Discussion

### 2.1 Fabrication and Assessment of nECTs

**FIGURE 2:**
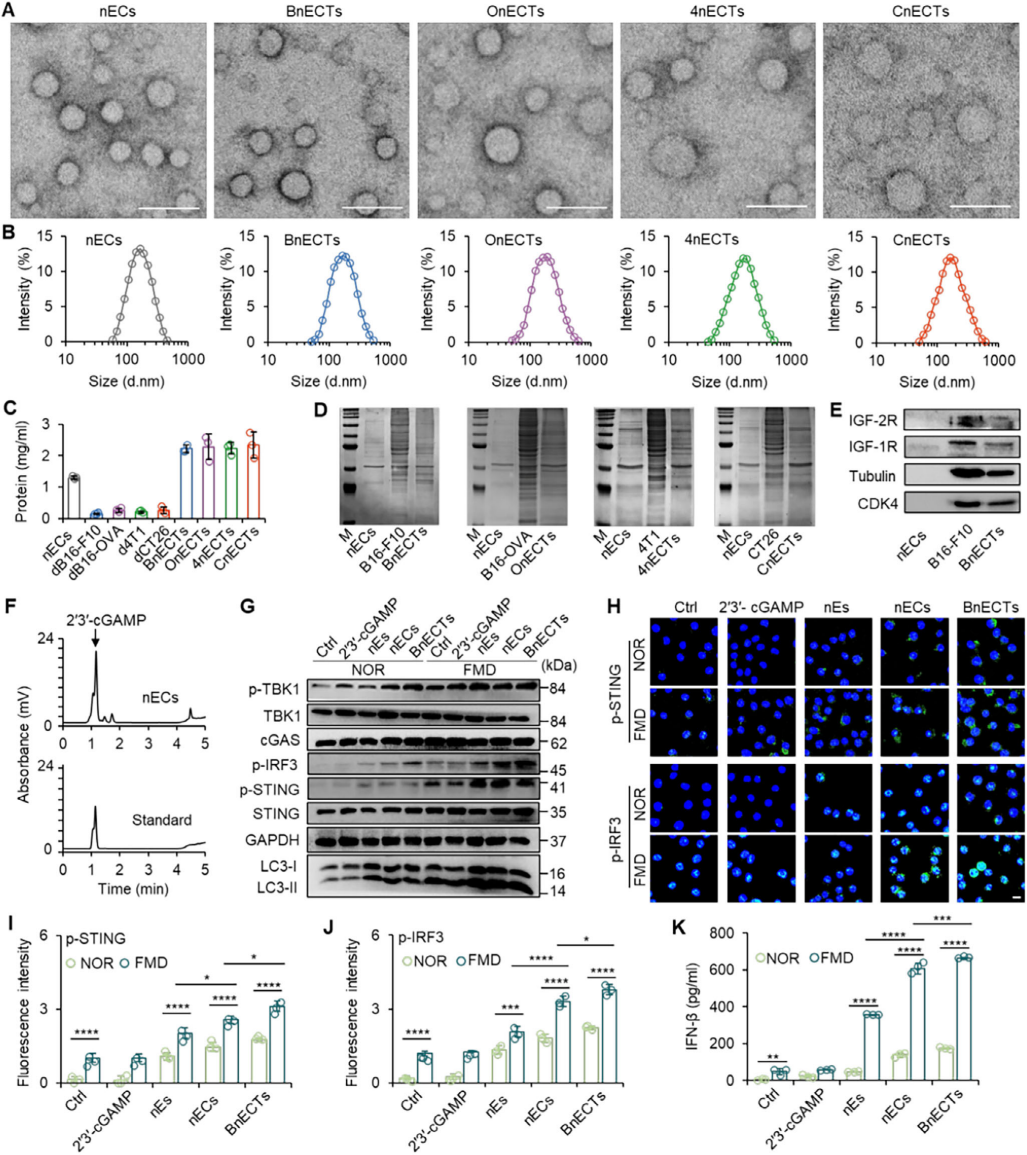
Preparation, characterization, and mediation of STING activation of nECTs. (A) TEM images of the nECs, BnECTs, OnECTs, 4nECTs, and CnECTs. Scale bars, 500 nm. (B) The size of nECs, BnECTs, OnECTs, 4nECTs, and CnECTs was determined by DLS at room temperature. (C) Protein concentrations of nECs, dB16‐F10, dB16‐OVA, d4T1, dCT26, BnECTs, OnECTs, 4nECTs, and CnECTs were determined using BCA protein assay. Error bars represent ±s.d. (*n* = 3). (D) SDS‐PAGE analysis of proteins in nECs, B16‐F10, B16‐OVA, 4T1, CT26, BnECTs, OnECTs, 4nECTs, and CnECTs. M, markers from 6.5 to 270 kDa. (E) Western blotting data of IGF‐2R, IGF‐1R, tubulin, and CDK4 in nECs, B16‐F10 tumor cells, and BnECTs. (F) The characteristic peaks of 2′3′‐cGAMP in the standard product and the extraction of nECs in the HPLC data. (G) Western blotting data of STING signaling pathway‐related proteins and LC3‐I/II in 2′3′‐cGAMP, nEs, nECs, and BnECTs‐treated DCs for 24 h. (H) Immunofluorescence staining of p‐STING and p‐IRF3 in DCs treated with 2′3′‐cGAMP, nEs, nECs, and BnECTs‐treated DCs for 24 h: scale bar, 10 µm. Quantitative analysis of (I) p‐STING and (J) p‐IRF3 in DCs treated with 2′3′‐cGAMP, nEs, nECs, and BnECTs treated DCs for 24 h. Error bars represent ± s.d. (*n* = 3). (K) Quantitative analysis of IFN‐β expressed in DCs cultured medium after treatment with 2′3′‐cGAMP, nEs, nECs, and BnECTs for 24 h. Error bars represent ± s.d. (*n* = 3). Statistical significance was assessed by one‐way ANOVA with Tukey test. *p < 0.05, **p < 0.01, ***p < 0.001, ****p < 0.0001.

The distinct 2′3′‐cGAMP synthesized by cGAS is essential for innate immune signaling in eukaryotes [42]. Using genetic engineering techniques (see Section 4 for details), the *E. coli* BL21 bacterial strain was engineered by incorporating the structural genes derived from m‐cGAS. m‐cGAS was produced in engineered bacteria when exposed to isopropyl β‐D‐1‐thiogalactopyranoside (IPTG) inducer, allowing for the stimulation and subsequent synthesis of 2'3'‐cGAMP (Figure S1). Western blot analysis reveals that cGAMP@*E.coli* achieves robust expression of cGAS proteins, whereas conventional *E. coli* exhibits no discernible expression of this protein. These findings confirm the successful expression of cGAS (Figure S2). The structure of *E. coli* and protoplasts expressing m‐cGAS was first studied with transmission electron microscopy (TEM), showing a classic rod‐like structure for *E. coli* and the spherical morphology of protoplasts after removing the cell wall (Figure S3). As previously examined, we subjected *E. coli* protoplasts to ultrasonic disruption, enabling their subsequent organization into nano‐sized assemblies (designated as nECs) with a mean diameter of around 190 nm. This dimension was confirmed through TEM imaging and quantified using dynamic light scattering (DLS) measurements. Next, the nECTs were fabricated by ultrasonic mixing of protoplasts of *E. coli* and whole tumor cells. The B16‐F10 melanoma cell line, along with its derivative B16‐OVA variant that expresses ovalbumin, 4T1 mammary tumor cells, or CT26 colon tumor cells, were mixed with protoplasts to synthesize the nECTs (BnECTs, OnECTs, 4nECTs, and CnECTs, respectively). The solution of nECTs exhibited the Tyndall effect, and spherical nanostructures were observed in the TEM images for all four kinds of nECTs (Figure 2A). The average size of nECTs was ≈200 nm, slightly larger than nECs (Figure 2B), and the surface potentials were negative, as shown in Figure S4. As shown in Figure S5, *E. coli* or protoplasts were quickly aggregated at the bottom of the tube, whereas no aggregates were observed in the nECs and nECTs solution. When a laser was shone, the light track could only be scattered and observed in the nECs and nECTs solution, confirming the formation of a colloidal solution.

To confirm the successful capture of tumor antigens, we first measured the total protein concentration in nECs or nECTs. As shown in Figure 2C, BnECTs, OnECTs, 4nECTs, or CnECTs contained more proteins than nECs, suggesting that proteins from tumor cells have been incorporated into the nECTs. Of note is that no nanoparticles could be collected if the tumor cells were treated directly with ultrasound. Few debris were collected with minimal proteins (dB16‐F10, dB16‐OVA, d4T1, or dCT26). The capture of tumor antigens in nECTs was then investigated by SDS‐PAGE. As shown in Figure 2D, most protein bands in B16‐F10, B16‐OVA, 4T1, or CT26 tumor cells were exhibited in the BnECTs, OnECTs, 4nECTs, or CnECTs, respectively. Western blots further showed that proteins from the tumor cell membrane, the receptors for insulin‐like growth factor 1 and 2 (IGF‐1R and IGF‐2R), cytoplasm (tubulin), and nuclei (cyclin‐dependent kinase 4, CDK4) were successfully captured in nECTs (Figure 2E). Notably, lipopolysaccharide (LPS) in the cell wall was removed while protoplasts were prepared. Therefore, the amount of LPS in nECs or nECTs was significantly lower than in *E. coli*, which can potentially reduce the undesirable immune responses (Figure S6). These data reveal that personalized bacteria‐based nECTs can capture whole tumor antigens for potential immunotherapy.

### 2.2 nECTs‐Mediated STING Activation

To determine the 2′3′‐cGAMP delivery of nECTs and to efficiently activate STING. Next, the existence of 2′3′‐cGAMP in nECs was confirmed with high‐performance liquid chromatography (HPLC), showing a similar peak compared with the standard samples (Figure 2F and Figure S7). The formation of nECTs did not affect the concentration of 2′3′‐cGAMP compared with nECs (Figure S8). In addition, mass spectrometry analysis revealed no significant structural differences between 2′3′‐cGAMP produced by *E. coli* and the standard 2′3′‐cGAMP, indicating that the 2′3′‐cGAMP was not affected by the intracellular environment of *E. coli* (Figure S9). The capability of nECTs to stimulate the STING signaling cascade was evaluated utilizing DCs. For this purpose, unmodified *E. coli* was employed to produce nEs, serving simultaneously as a baseline comparator. Following treatment with either free 2′3′‐cGAMP, nEs, nECs, or BnECTs, we probed the expression of related proteins integral to this STING pathway, phosphorylated IRF3 (p‐IRF3), phosphorylated TBK1 (p‐TBK1), and phosphorylated STING (p‐STING), as well as the secretion of IFN‐β in DCs through Western blot analysis. As shown in Figure 2G, various therapies have minimal effects on the expression of cGAS in DCs. The p‐STING was slightly upregulated in nEs, nECs, and BnECTs treated DCs cultured in a standard medium. Interestingly, the expressions of p‐STING were enormously increased when culturing DCs in an FMD medium with nECs or nECTs. Similarly, p‐TBK1 was upregulated in nEs, nECs, and BnECTs‐treated DCs. Both p‐TBK1 and STING can interact with IRF3 and induce the p‐IRF3. Western blot data confirmed that p‐IRF3 was upregulated in the nEC group, while BnECT treatment exhibited the highest expression of p‐IRF3 when combined with FMD treatment. These results were further validated with immunofluorescence staining, confirming that the existence of 2′3′‐cGAMP in nECs could increase the amount of p‐STING and p‐IRF3 in DCs, and the highest fluorescence intensity was obtained in BnECTs groups (Figure 2H–J). FMD treatment promoted the production of p‐STING or p‐IRF3 in DCs, while free 2′3′‐cGAMP treatment had minimal effects on the p‐STING or p‐IRF3 expression (Figure 2H–J).

An essential feature of the STING pathway is the activation of autophagy derived from LC3 lipidation, which is crucial in enhancing innate immunity [43]. Meanwhile, ubiquitination is also involved in the STING pathway [44]. The ubiquitinated STING can promote its translocation and phosphorylation, essential for regulating STING/TBK1 trafficking and rendezvousing with IRF3 [45]. Interestingly, we observed that FMD treatment not only upregulated LC3 expression but also enhanced LC3 lipidation, further promoting endoplasmic reticulum (ER) stress [46, 47]. Investigation of ubiquitinated proteins showed that FMD treatment increased the ubiquitinated proteins in DCs (Figure S10). Specifically, the protein band at 35 kDa should belong to STING, which was enhanced under FMD conditions. Consequently, the secretion of IFN‐β was minimal in the control or free 2′3′‐cGAMP group, because the stability of 2′3′‐cGAMP markedly diminishes in the presence of pyrophosphatase and phosphodiesterase [48]. In addition, owing to its hydrophilic and negatively charged characteristics, 2′3′‐cGAMP demonstrates inefficient penetration through the lipid bilayer of cellular membranes [49]. Treatment of nECs triggered higher secretion of IFN‐β than nEs, and BnECTs induced the highest concentration (Figure 2K). In particular, FMD treatment dramatically enhanced the secretion of IFN‐β in nEs, nECs, or BnECTs‐treated DCs. Similarly, BnECT treatment showed the highest level of IFN‐β (Figure 2K). Together, these data confirm that effective delivery of 2′3′‐cGAMP by nECTs combined with FMD was essential for the activation of STING.

### 2.3 nECTs‐Mediated Immunostimulatory Potency and Antigen Cross‐Presentation

**FIGURE 3:**
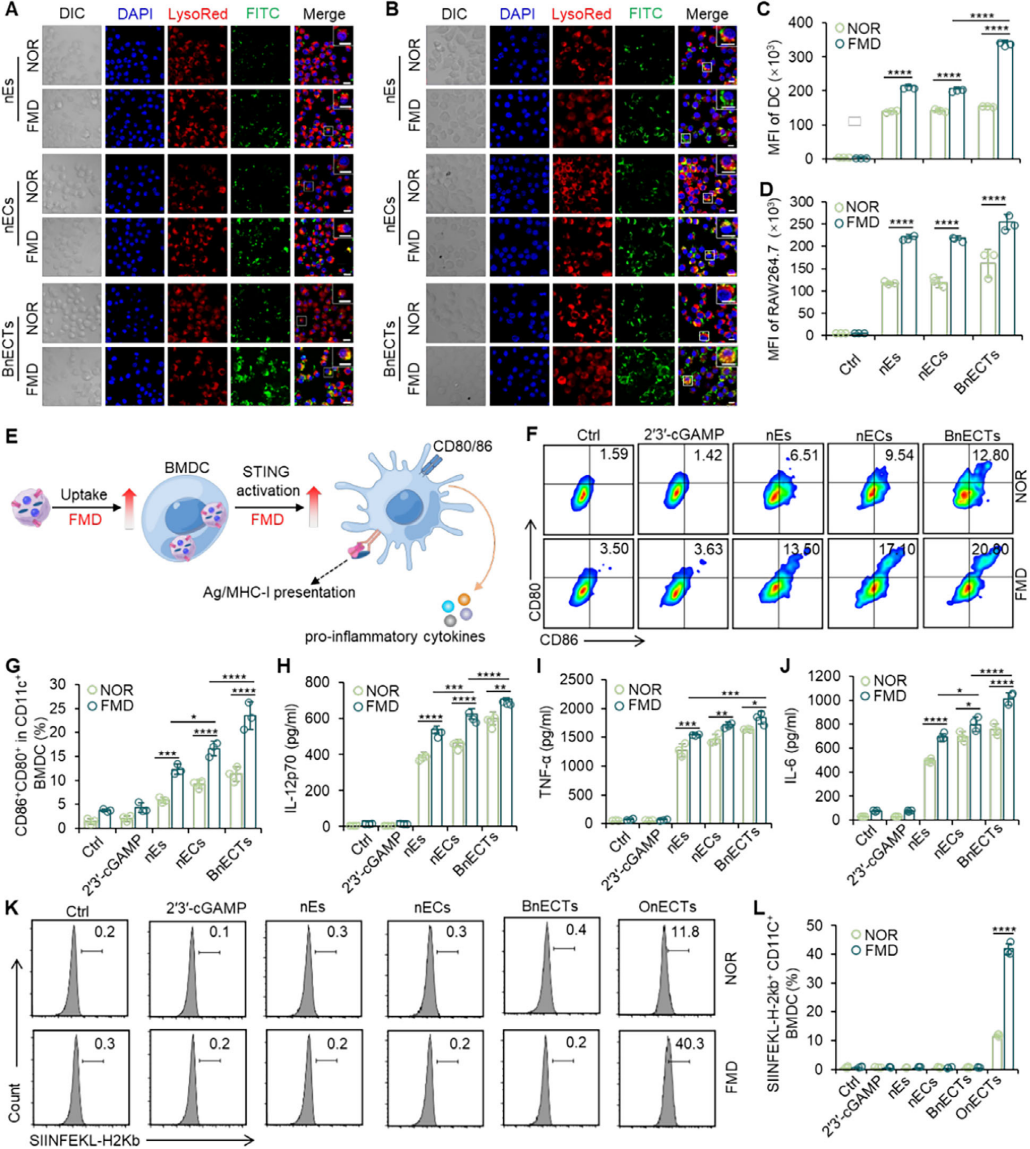
In vitro activation of APCs and antigen cross‐presentation. Confocal images of (A) DCs and (B) RAW264.7 were treated with FITC‐labelled nEs, nECs, or BnECTs for 6 h. The nuclei and endo/lysosomes were stained with DAPI and LysoTracker Red (LysoRed), respectively. Scale bar, 10 µm. Mean fluorescence intensity (MFI) in (C) DCs and (D) RAW264.7 treated with FITC‐labelled nEs, nECs, or BnECTs. (E) Schematic illustration of nECTs‐induced DC activation and tumor antigen presentation. (F) Representative flow cytometry data and (G) quantitative analysis of CD86^+^CD80^+^ BMDCs with various treatments for 24 h. Quantitative analysis of (H) IL‐12p70, (I) TNF‐α, and (J) IL‐6 secreted by BMDCs with various treatments for 24 h. (K) Representative flow cytometry data and (L) quantitative analysis of SIINFEKL peptide‐positive BMDCs with various treatments for 24 h. Error bars represent ± s.d. (*n* = 3). Statistical significance was assessed by one‐way ANOVA with Tukey test. *p < 0.05, **p < 0.01, ***p < 0.001, ****p < 0.0001.

As shown in Figure 3A–D (for 6 h) and Figure S11A–F (for 2 h), confocal images and flow cytometry data showed that DCs and RAW264.7 could capture fluorescein isothiocyanate (FITC)‐labeled nEs, nECs or BnECTs efficiently. Especially under FMD conditions, there was a substantial rise in the internalization of BnECTs within DCs and RAW264.7 cells, suggesting that FMD administration boosts the cellular incorporation of nECTs. The fusion process of BnECTs enhances the density and diversity of tumor‐associated antigens (TAAs) on the surface of the nanoparticles, making them more recognizable to APCs. As a result, the increased visibility of these TAAs to APCs leads to a higher probability of recognition and capture, thereby improving the overall uptake efficiency. For the comparison of nECs and BnECTs, the fusion process of BnECTs enhances the density and diversity of TAAs on the surface of the nanoparticles, making them more recognizable to APCs [50]. As a result, the increased visibility of these TAAs to APCs leads to a higher probability of recognition and capture, thereby improving the overall uptake efficiency. Consequently, engaging in the FMD exposes cells to an environment with lower glucose and serum concentrations. Research has shown that DCs grown under conditions of reduced glucose, such as a concentration of 1% glucose, and with restricted serum availability, can elevate their responsiveness to external stimuli, such as nanoparticles [51]. Regarding RAW264.7 cells, they are capable of ingesting pathogens or other foreign substances through phagocytosis and subsequently degrading these materials into smaller peptides within their cellular compartments as crucial antigen‐presenting cells. Following this degradation, the peptides bind to MHC molecules and are transported to the cell surface, forming complexes that can be recognized by receptors on the surface of T cells. This recognition process activates T cells and initiates adaptive immune responses. Moreover, macrophages secrete a variety of cytokines, which further modulate and potentiate immune reactions, ensuring effective clearance of invaders while maintaining immunological homeostasis [52].

Subsequently, the stimulation of APCs was explored utilizing bone marrow‐derived dendritic cells (BMDCs) (Figure 3E). These cells were subjected to incubation with unbound 2′3′‐cGAMP, nEs, nECs, or BnECTs, employing either standard or FMD‐conditioned media for a duration of 24 h. Subsequent analysis was conducted to evaluate the expression levels of co‐stimulatory markers, specifically for CD80 and CD86, showing BnECTs or nECs induced the maturation of BMDCs more efficiently than free 2′3′‐cGAMP or nEs (Figure 3F,G and Figure S12). Notably, the percentages of CD86^+^CD80^+^ BMDCs were significantly increased when cultured in an FMD medium. Consistent with the activation of the STING pathway, this suggests that nECTs efficiently deliver 2′3′‐cGAMP to stimulate STING to further enhance DCs maturation.

Besides surface expression of co‐stimulatory molecules, DC maturation also involves the redistribution of MHC molecules from the intracellular to the cell surface and the secretion of various cytokines [53, 54]. As shown in Figure S13A–C, the surface density of MHC‐I molecules was quantitatively assessed on BMDCs in the nEs, nECs, and BnECTs groups and was increased compared with the free 2′3′‐cGAMP treatment, and interestingly, the BnECTs group was upregulated most significantly. Similarly, FMD treatment significantly increased the proportion of MHC‐I^+^ BMDCs. Detection of cytokines confirmed that nEs, nECs, or BnECTs promoted BMDCs to secrete more interleukin‐6 (IL‐6), and tumor necrosis factor‐α (TNF‐α), and IL‐12p70 than other treatments (Figure 3H–J). Nanoparticles (nanovaccine) can serve as efficient carriers to facilitate the cross‐presentation of protein fragments to MHC‐I. Specifically, nanoparticles can be taken up by antigen‐presenting cells, such as dendritic cells, via endocytosis. Once inside the cell, the protein fragments carried by the nanoparticles can escape from endosomes or lysosomes into the cytoplasm through a retrograde transport pathway. In the cytoplasm, these exogenous protein fragments can be degraded into short peptides by proteasomes and then transported into the endoplasmic reticulum via the transporter associated with antigen processing, where they bind to newly synthesized MHC‐I molecules to form complexes. Finally, these complexes are transported to the cell surface, thereby achieving the cross‐presentation of exogenous protein fragments via MHC‐I molecules, which stimulate specific CD8^+^ T cell immune responses [55, 56]. This process not only enhances the efficiency of antigen recognition by the immune system but also improves the efficacy of vaccines or immunotherapies. Therefore, to verify that our nanovaccine facilitates the presentation of a specific tumor antigen through the MHC‐I pathway, we conducted an assay to assess the binding of the peptide SIIQFEKL to the H‐2Kb molecule. Figures 3K,L and Figure S14 shows that the OVA presentation could only be detected in OnECTs‐treated BMDCs. FMD treatment further increased the MHC‐I presentation of OVA, which confirmed the successful presentation of tumor‐derived peptides. The findings indicate that nECTs can better deliver 2′3′‐cGAMP to trigger STING activation and promote DCs maturation to enhance antigen presentation; on the contrary, free 2′3′‐cGAMP failed to touch STING activation. Interestingly, FMD treatment is a powerful adjunctive method for triggering an immune response.

### 2.4 Intralymphatic Delivery of nECTs for Activating Adaptive Immunity

**FIGURE 4:**
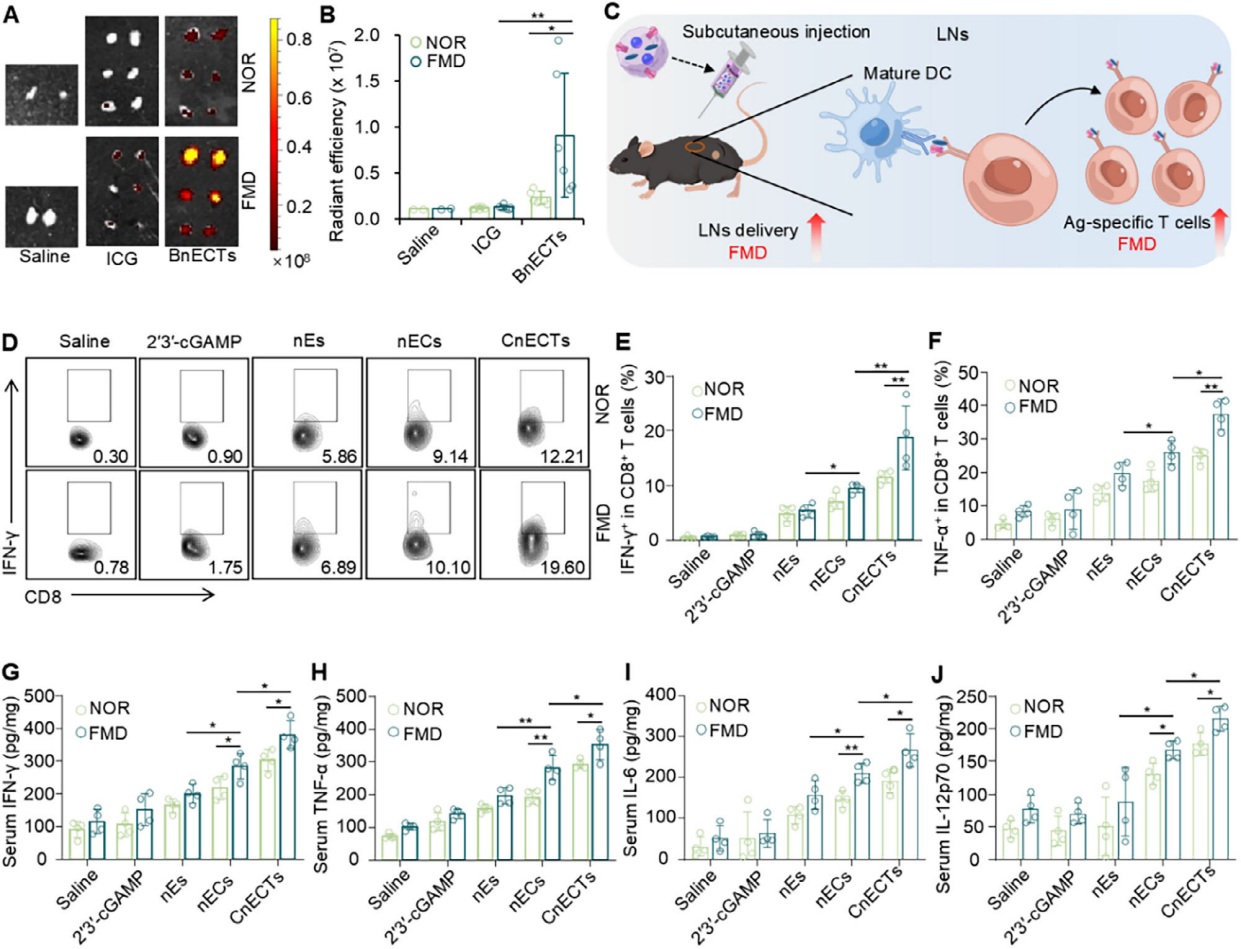
nECTs combined with FMD for lymph node targeting and stimulation of adaptive immune responses in vivo. (A) Ex vivo IVIS imaging and (B) quantitative analysis of fluorescence intensity in draining lymph nodes after treatment with free ICG or ICG‐labelled BnECTs for 24 h. (C) Schematic illustration of antigen‐specific T cell response stimulation using nECTs with FMD treatment. (D) Flow cytometry data and (E) quantitative analysis of IFN‐γ‐positive cells in CD3^+^CD8^+^ T cells in lymph nodes on Day 22 post‐immunization of BALB/C mice. (F) Quantitative analysis of TNF‐α‐positive cells in CD3^+^CD8^+^ T cells in lymph nodes on Day 22 post‐immunization of BALB/C mice. Quantitative analysis of (G) IFN‐γ, (H) TNF‐α, (I) IL‐6, and (J) IL‐12p70 in the serum of the BALB/C mice with various treatments. Error bars represent ± s.d. (*n* = 4). Statistical significance was assessed by one‐way ANOVA with Tukey test. *p < 0.05, **p < 0.01.

The animal studies conducted for this research complied fully with both the legal requirements and the ethical guidelines established by our institution's Animal Care and Use Committee at the National Center for Nanoscience and Technology (NCNST21‐2010‐15). To assess in vivo lymphatic delivery, fluorescently tagged BnECTs were administered via subcutaneous injection in C57BL/6 mice. For in vivo FMD treatment, a dietary regimen consisting of a 50% calorie decrease on the initial day, succeeded by a 90% calorie restriction for the subsequent pair of days, was imposed on C57BL/6 mice, and imaging of the major organs and LNs was conducted 24 h post‐injection. Figure 4A,B and Figure S15 illustrate that FMD therapy significantly enhanced the concentration of BnECTs in the LNs when juxtaposed with mice consuming a standard diet. However, the FMD treatment did not exert a substantial impact on the dissemination of BnECTs throughout the primary organs. FMD has shown potential in enhancing the cellular uptake of vaccines by dendritic cells [51], thereby promoting the activation of these cells around lymph nodes. This enhancement could result in a greater number of dendritic cells migrating to lymph nodes, which in turn amplifies the immune response. Additionally, when the body transitions into an energy‐conservation mode, it may augment the efficiency of both blood and lymphatic circulatory systems to optimize the utilization of limited resources. Thus, brief periods of FMDs could boost lymphatic circulation, which aids in the swift delivery of vaccine constituents to lymph nodes.

To understand the mechanisms associated with the antitumor activity of nECTs (Figure 4C), we analyzed adaptive immune cell populations in LNs from mice bearing B16‐F10 tumors at the three‐week mark and mice bearing CT26 tumors 2 days later. Next, we examined the immune reactions in BALB/c or C57BL/6 mice vaccinated with CnECTs or OnECTs, respectively. Probably due to the better activation of DCs and accumulation in LNs, after vaccination with CnECTs (Figure 4D–F) or OnECTs (Figure S16A,B) under FMD conditions, there was a significant rise in the population of IFN‐γ or TNF‐α expressing CD8^+^ T cells within the LNs. Following immunization, the body initiates an immune response to the antigenic components of the vaccine by first activating naive T cells. These cells subsequently undergo proliferation and differentiation into effector T cells [57, 58]. Detection of OVA IgG in serum also confirmed the successful cross‐presentation of OVA antigen in OnECTs vaccinated mice (Figure S17). An appreciable increase was recorded in the serum levels of various pro‐inflammatory cytokines, encompassing IFN‐γ, IL‐6, TNF‐α, and IL‐12p70 in BALB/c mice vaccinated with nEs, nECs, and CnECTs compared to the 2′3′‐cGAMP group (Figure 4G–J) where they were higher in the nECs and CnECTs groups than in the nECs. These data suggest that nECTs could effectively accumulate in LNs, promote T cell proliferation, and further enhance the immune system by FMD treatment.

### 2.5 Anti‐Melanoma Tumor Treatment and Immunity

**FIGURE 5:**
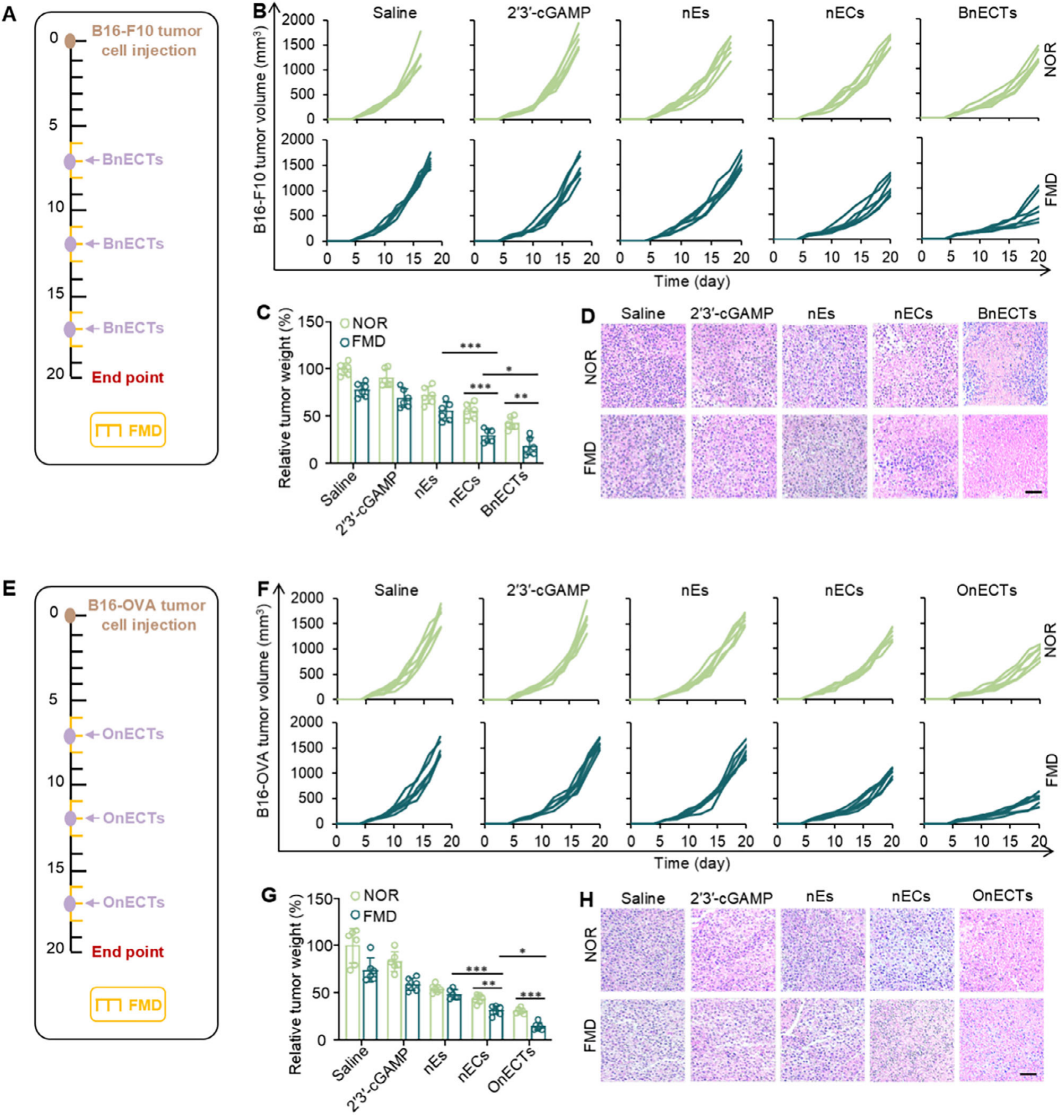
nECTs‐mediated anti‐tumor effects in murine melanoma models. (A) In vivo treatment schedule of B16‐F10 tumor models. (B) Individual tumor growth curves of B16‐F10 tumors with various treatments. (C) Tumor weights of B16‐F10 tumors. Error bars represent ± s.d. (*n* = 6). (D) H&E images of B16‐F10 tumors. Scale bar, 50 µm. (E) In vivo treatment schedule of B16‐OVA tumor models. (F) Individual tumor growth curves of B16‐OVA tumors with various treatments. (G) Tumor weights of B16‐OVA tumors. Error bars represent ± s.d. (*n* = 6). (H) H&E images of B16‐OVA tumors. Scale bar, 50 µm. Statistical significance was assessed by one‐way ANOVA with the Tukey test. *p < 0.05, **p < 0.01, ***p < 0.001.

Given the robust accumulation of nECTs in LNs and the activation of immune cell populations, we investigated the contribution of nECTs to the generation of tumor‐suppressing effectiveness. Treatment with BnECTs was administered to mice bearing B16‐F10 tumors on Days 7, 12, and 17 (Figure 5A). As shown in Figure 5B and Figure S18, BnECTs inhibited tumor growth better than nECs, suggesting the importance of capturing tumor antigens. Importantly, nECs exhibited better anti‐tumor capacity than nEs. As shown in Figure 5C, tumor weights verified that nECTs‐mediated STING activation effectively inhibited B16‐F10 tumor growth. H&E images showed extensive apoptosis in nECTs‐treated tumors, especially under FMD conditions (Figure 5D). Based on the above role shaping, we inferred that nECTs could be used as a personalized vaccine for cancer vaccine development. To test this hypothesis, we evaluated the anti‐melanoma capacity of OnECTs using B16‐OVA melanoma models. Treatment with OnECTs was given to mice that were models for B16‐OVA tumor development on Days 7, 12, and 17 (Figure 5E). As shown in Figure 5F and Figure S19, OnECTs inhibited tumor growth better than nECs, suggesting the importance of capturing tumor antigens. As shown in Figure 5G, tumor weights supported the idea that capturing and cross‐presenting tumor antigens could induce better anti‐tumor immunity. Interestingly, the antitumor effect of nECs was superior to nEs, demonstrating the importance of effective delivery of 2′3′‐cGAMP by nECs and OnECTs. H&E images demonstrated widespread apoptosis in the tumors post‐treatment with nECTs, especially under FMD conditions (Figure 5H).

Within the tumor microenvironment, a sophisticated network of immune cells is established, encompassing a diverse array of cell types including T lymphocytes, B lymphocytes, NK cells, macrophages, and dendritic cells. Despite this diversity, practical immune cell profiling within the TME often necessitates the selective detection of specific immune cell subsets. Within the realm of adaptive immunotherapy, the identification of T cells within the TME assumes particular importance owing to their pivotal role in the direct recognition and eradication of neoplastic cells [59, 60]. Through the meticulous examination of T cell infiltration levels, phenotypic traits, and functional states, a more profound comprehension of the TME can be attained. Consequently, this investigation prioritizes the quantification and functional assessment of CD8^+^ cytotoxic T lymphocytes. Beyond the emphasis on T cells, alterations in other immune cell populations have also been documented, notably an augmentation in dendritic cell presence and a diminution in M2 macrophage numbers, which provide valuable ancillary data. As depicted in Figure S20A, both nECs and BnECTs triggered a significant increase in the count of cytotoxic T lymphocytes within B16‐F10 melanoma masses. The combination of FMD and BnECTs led to a superior induction of CD8^+^ cytotoxic T cells in B16‐F10 tumors compared to other experimental arms. Immunization using BnECTs resulted in a greater proportion of IFN‐γ or TNF‐α secreting CD8^+^ T cells in B16‐F10 tumors, as evidenced by Figure S20B–E. An in‐depth analysis of the tumor's immune milieu revealed a decline in tumor‐associated macrophages (identified by CD206 expression on F4/80^+^CD11b^+^ cells) following BnECT treatment of B16‐F10 tumors (Figure S21A). Conversely, BnECTs enhanced the presence of MHC‐II^+^CD11c^+^ cells and natural killer (NK) cells within B16‐F10 tumors, as illustrated in Figure S21B,C. Detection of cytokines showed that nECs activated a better immune response than nEs, but tumor antigens captured BnECTs exhibited the highest concentrations of cytokines in B16‐F10 tumors (Figure S22A–D). Similarly, immunofluorescence staining analyzed the number of tumors infiltrating lymphocytes in B16‐OVA tumor tissues. Corroborating the observations of CD8^+^ T cell infiltration in B16‐OVA tumors, OnECTs group exhibited a pronounced increase in CD8^+^ T cell density compared to all other tested groups, as demonstrated in Figure S23A. Moreover, our investigations revealed an augmented fraction of IFN‐γ or TNF‐α secreting CD8^+^ T cells in B16‐OVA tumors following treatment with OnECTs, compared with equivalent doses of free 2′3′‐cGAMP or untreated mice (Figure S23B–E). The immune microenvironment of B16‐OVA tumors was then analyzed, showing tumor‐associated macrophages (CD206^+^ in F4/80^+^CD11b^+^) were decreased in B16‐OVA tumors after treatments with OnECTs (Figure S24A). By contrast, OnECTs increased the amount of MHC‐II^+^CD11c^+^ and NK cells in the B16‐OVA tumor model (Figure 24B,C). Detection of cytokines showed that nECs activated a better immune response than nEs, but tumor antigens captured by OnECTs exhibited the highest concentrations of cytokines in B16‐F10 tumors (Figure S25A–D).

To check the STING signal pathway, the p‐IRF3‐positive cells in tumors showed the highest amount in nECTs and FMD‐treated tumors (Figure S26A,B). The amount of IFN‐β in tumors was obviously increased in the OnECTs group (Figure S26C). Previous investigations have indicated that IFN‐β might be critical in the natural activation of tumor‐reactive CD8^+^ T lymphocytes. Consequently, staining p‐STING in tumor tissues also confirmed the upregulation of p‐STING in OnECTs‐treated tumors (Figure S27A,B).

The safety profile associated with nECT‐induced STING stimulation was subjected to further investigation. Subsequently, administering OnECTs did not induce any notable alterations in the body weight of experimental mice throughout the duration of the in vivo trials (Figure S28). Feeding mice an FMD diet resulted in a decrease in body weight; however, this reduction was transient as weight normalized upon resumption of a standard diet (Figure S28). Post‐experimental dissection and histological examination of vital organs through H&E staining revealed no evidence of substantial organ impairment or inflammation in C57BL/6 mice that had undergone vaccination with nECTs and subsequent FMD interventions (Figure S29). Furthermore, biochemical markers indicative of liver and cardiac health, namely alanine aminotransferase, alkaline phosphatase, aspartate aminotransferase, and creatine kinase (CK), remained within normal ranges, suggesting that nECT and FMD treatments did not compromise these organ functions (Figure S30). The immunotherapy may lead to cytokine storm, contributing to hyperinflammation‐ and hypercoagulation‐driven mortality. As shown in Figure S31, IgG in serum was slightly increased with nECTs treatment, and no apparent changes were observed in IgG2c. Together, our findings illustrate that the therapeutic approach involving nECTs elicits the most potent immunological response against tumors. This is achieved through the induction of STING pathway activation, which in turn promotes the maturation of DCs. Consequently, there is a surge in the generation of pro‐inflammatory cytokines, alongside the enhancement of CD8^+^ T cell differentiation and expansion, thereby amplifying the body's defense mechanisms against malignancies and resulting in the most effective tumor suppression.

### 2.6 Anti‐Colorectal and Breast Tumor Treatment and Immunity

**FIGURE 6:**
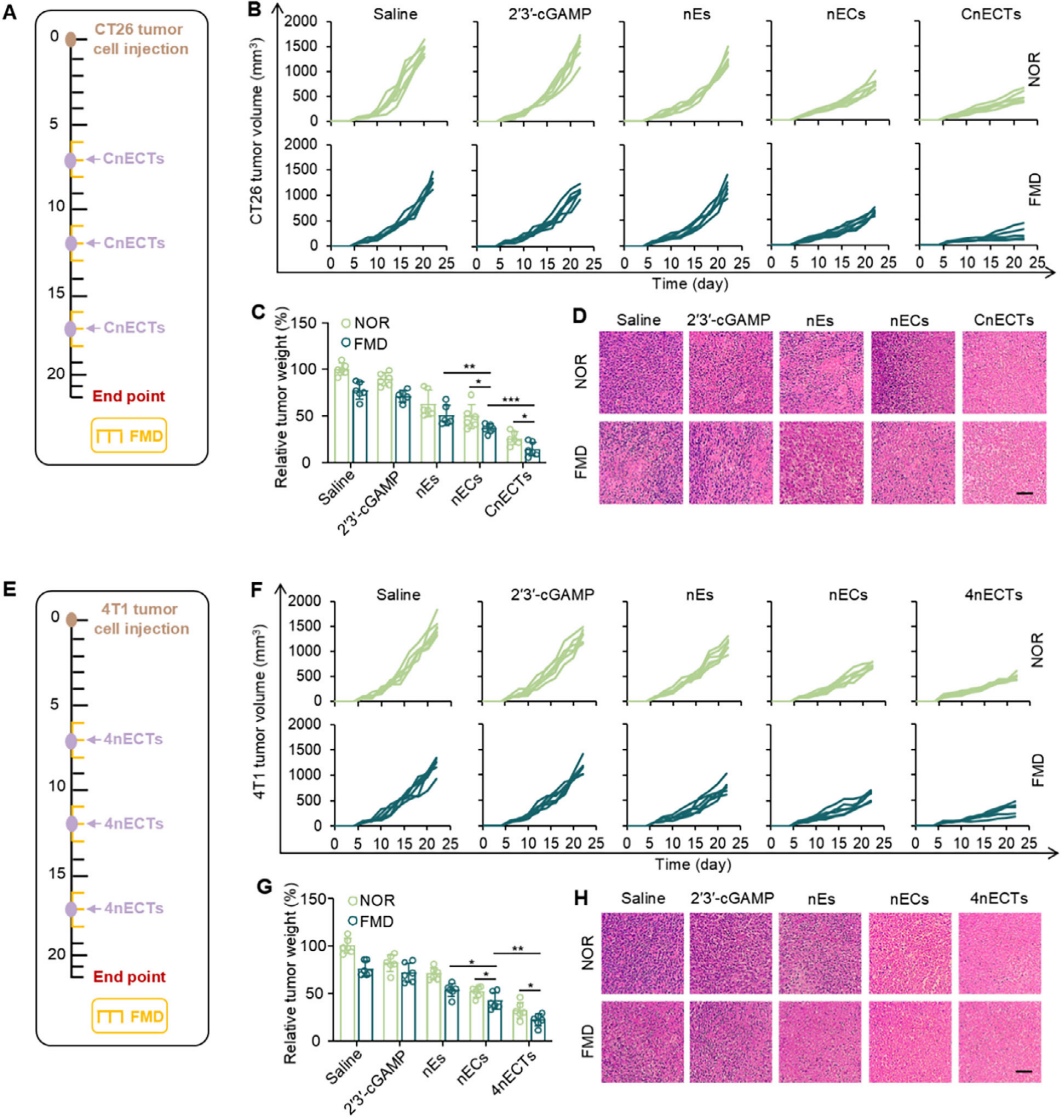
nECTs‐mediated anti‐tumor effects in murine melanoma models. (A) In vivo treatment schedule of CT26 tumor models. (B) Individual tumor growth curves of CT26 tumors with various treatments. (C) Tumor weights of CT26 tumors. Error bars represent ± s.d. (*n* = 6). (D) H&E images of CT26 tumors. Scale bar, 50 µm. (E) In vivo treatment schedule of 4T1 tumor models. (F) Individual tumor growth curves of 4T1 tumors with various treatments. (G) Tumor weights of 4T1 tumors. Error bars represent ± s.d. (*n* = 6). (H) H&E images of 4T1 tumors. Scale bar, 50 µm. Statistical significance was assessed by one‐way ANOVA with the Tukey test. *p < 0.05, **p < 0.01, ***p < 0.001.

CT26 Tumors are generally considered non‐inflammatory tumor types, and most colorectal cancers are not responsive to checkpoint‐blocking antibody therapy [61]. To test whether nECTs could stimulate anti‐tumor immune responses in colorectal cancer patients, we prepared colorectal cancer‐specific vaccines as CnECTs. We established tumor models using CT26 tumor cells and treated mice with saline, free 2′3′‐cGAMP, nEs, nECs, and CnECTs on Days 7, 12, and 17 (Figure 6A). FMD treatment was conducted as abovementioned. As shown in Figure 6B and Figure S32, free 2′3′‐cGAMP or nEs slightly decreased the tumor growth of CT26 tumors. In contrast, nECs or CnECTs efficiently inhibited tumor growth, probably due to the stimulation of the immune system via triggering the STING signaling pathway. In particular, CnECTs exhibited the best antitumor capacity, revealing the importance of capturing and cross‐priming specific tumor antigens. Interestingly, FMD treatment further decreased the tumor growth compared with the normal group. The therapeutic results were validated with tumor weights, confirming that CnECTs exhibited the best anti‐tumor results with or without FMD treatment, further confirming the importance of capturing tumor antigens (Figure 6C). It is worth noting that treatment of free 2′3′‐cGAMP did not affect the tumor growth but was significantly improved with nECs, suggesting the importance of targeted delivery of STING agonists. Histological analysis also confirmed extensive apoptosis in CnECTs treated CT26 tumors (Figure 6D). Breast cancer is generally considered to be another non‐inflammatory, and breast cancer patients do not respond well to treatment with checkpoint‐blocking antibodies [62]. We also established tumor models using 4T1 cells and treated mice with saline, free 2′3′‐cGAMP, nEs, nECs, and 4nECTs on Days 7, 12, and 17 (Figure 6E). Similarly, 4nECTs exhibited the best tumor growth inhibitory effect with or without FMD treatment (Figure 6F and Figure S33). Meanwhile, treatment with free 2′3′‐cGAMP did not affect tumor growth, but the use of nECs still significantly inhibited tumor growth, suggesting the importance of targeted delivery of STING agonists. Tumor weights further validated previous treatment results (Figure 6G). Histological analysis also confirmed that 4T1 tumors treated with 4nECTs showed extensive apoptosis (Figure 6H). Significantly, FMD further enhances the anti‐tumor effect. In summary, the ability of nECTs to efficiently deliver 2′3′‐cGAMP and tumor‐associated antigens can lead to regression of established CT26 and 4T1 tumors and synergistically provide a potent antitumor effect with FMD therapy.

The biosafety of nECTs or FMD treatment was evaluated during in vivo treatments. As shown in Figure S34, nECTs have minimal effects on the body weights of mice consuming standard diets. The application of FMD was observed to induce a transient reduction in body weight among the subjects, which rapidly normalized upon resuming a standard diet regimen. Consistently, fluctuations in blood glucose levels mirrored this pattern, with a noticeable decrease during the FMD phase followed by a swift return to baseline values when normal dietary intake was restored (Figure S35). H&E staining of vital organs, heart, lung, liver, spleen, and kidney, did not exhibit any substantial signs of tissue damage or adverse effects resulting from the FMD intervention (Figure S36). This indicates that the metabolic changes induced by the FMD did not compromise the structural integrity or functional health of these critical organs. Moreover, blood biochemistry tests showed no significant differences between the saline group and FMD or FMD with nECTs‐treated mice (Figure S37). These data indicate that the FMD or nECT treatment should not induce apparent side effects during treatment.

**FIGURE 7:**
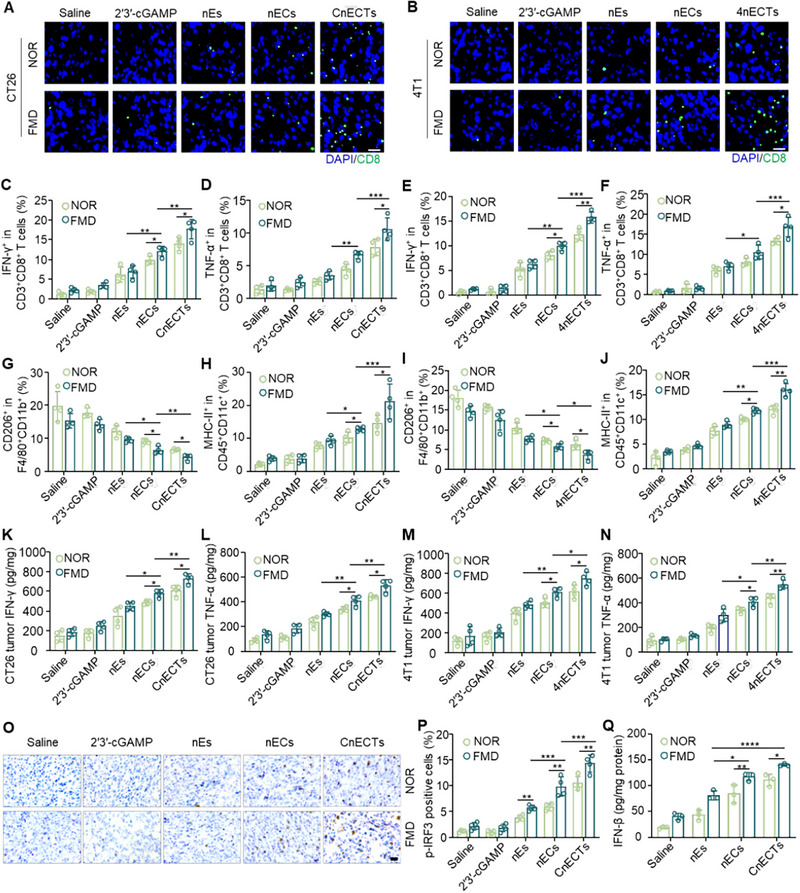
In vivo mechanistic study of nECTs‐mediated antitumor immunity. (A) Confocal images of infiltrated CD8^+^ T cells in the CT26 tumors. Scale bar, 50 µm. (B) Confocal images of infiltrated CD8^+^ T cells in the 4T1 tumors. Scale bar, 50 µm. Quantitative analysis of (C) IFN‐γ‐positive cells and (D) TNF‐α‐positive cells in CD3^+^CD8^+^ T cells in CT26 tumors. Error bars represent ± s.d. (*n* = 4). Quantitative analysis of (E) IFN‐γ‐positive cells and (F) TNF‐α‐positive cells in CD3^+^CD8^+^ T cells in 4T1 tumors. Error bars represent ± s.d. (*n* = 4). Quantitative analysis of (G) CD206‐positive macrophages and (H) MHC‐II‐positive DCs in CT26 tumors. Error bars represent ± s.d. (*n* = 4). Quantitative analysis of (I) CD206‐positive macrophages and (J) MHC‐II‐positive DCs in 4T1 tumors. Error bars represent ± s.d. (*n* = 4). Quantitative analysis of the (K) IFN‐γ and (L) TNF‐α in the CT26 tumors measured by ELISA kits. Error bars represent ± s.d. (*n* = 4). Quantitative analysis of the (M) IFN‐γ and (N) TNF‐α in the 4T1 tumors measured by ELISA kits. Error bars represent ± s.d. (*n* = 4). (O) Immunohistochemical images of p‐IRF3 expressed cells (brown) in CT26 tumor tissues. Scale bar, 50 µm. (P) Quantitative analysis of p‐IRF3 positive cells in the CT26 tumors. Error bars represent ± s.d. (*n* = 4). (Q) Quantitative analysis of IFN‐β in the CT26 tumors. Error bars represent ± s.d. (*n* = 3). Statistical significance was assessed by one‐way ANOVA with Tukey test. *p < 0.05, **p < 0.01, ***p < 0.001, ****p < 0.0001.

Immunofluorescence staining and flow cytometry were also used to analyze the number and phenotyping of TILs and their associated pro‐inflammatory factors in CT26 and 4T1 tumor tissues. The spatial distribution of CD8^+^ T lymphocytes within CT26 and 4T1 tumor microenvironments was quantified using immunofluorescence techniques. As depicted in Figure 7A,B, the density of CD8^+^ T cells notably escalated in tumors subjected to CnECTs and 4nECTs therapy, particularly when administered in conjunction with FMD, signifying an enhanced immune infiltration. Furthermore, the administration of either CnECTs or 4nECTs as vaccinations prompted an obvious elevation in the proportion of IFN‐γ or TNF‐α secreting CD8^+^ T cells within CT26 tumors and 4T1 models, respectively. These findings are illustrated in Figure 7C–F, highlighting the potential of these treatments in stimulating a robust antitumor immune response characterized by heightened cytokine secretion.

We also further characterized the immune microenvironment of CT26 tumors. As shown in Figure 7G, following treatment with nECs or CnECTs under FMD conditions, there was a notable diminution in the population of tumor‐promoting macrophages, specifically those expressing CD206 within the F4/80 and CD11b positive subset [63], which are known to facilitate cancer advancement and suppress immune responses against tumors. Moreover, CnECTs increased the amount of tumor‐infiltrating MHC‐II^+^ CD11c^+^ cells in CT26 tumors (Figure 7H), which can stimulate CD4^+^ T cells, thereby amplifying cytotoxic capabilities of CD8^+^ T cells [64]. The appeal results are likewise reflected in the 4T1 tumor (Figure 7I,J). Detections of key inflammatory mediators, encompassing IFN‐γ, IL‐6, TNF‐α, and IL‐12p70, confirmed that nECTs could augment the secretion of these cytokines in CT26 and 4T1 (Figure 7K–N, Figures S38 and S39). These data suggest that nECTs can reshape the tumor immunosuppressive microenvironment.

Major immune processes such as STING activation and cytokine secretion were subsequently studied and compared to achieve a holistic insight into the robust anti‐tumoral immunity elicited by immunotherapies mediated through nECTs. The expression profiles of proteins linked to the STING pathway within tumor tissue samples were assessed utilizing immunohistochemistry specific to this pathway. Next, p‐IRF3‐positive cells in CT26 tumors were analyzed. As shown in Figures 7O–P, there was a remarkable escalation in the quantity of p‐IRF3 expressing cells in tumors that had been subjected to treatment with CnECTs. Detection of IFN‐β also confirmed the enhanced secretions in tumors vaccinated with nECs or CnECTs (Figure 7Q). The findings imply that the therapeutic approach facilitated by nECTs instigates the most potent anti‐tumor immune reaction through the induction of STING pathway activation, enhancement of tumor‐associated antigen presentation, and the promotion of pro‐inflammatory cytokine secretion. Concurrent synergistic FMD therapy triggered the highest anti‐tumor immune response.

## 3 Conclusion

In summary, we developed a general platform to fabricate personalized nanovaccines capable of activating adaptive and innate immunity for combating tumors. Bacteria expressing m‐cGAS generated a nanosized structure and captured whole autologous tumor antigens using a simple ultrasound mixing method instead of complicated chemical synthesis. Compared with immune checkpoint inhibitors, nECTs precisely activate the STING signaling pathway, promoting the activation of dendritic cells and the cross‐presentation of antigens, which primarily induces localized immune activation. This localized activation can potentially mitigate systemic side effects. Moreover, nECTs utilize the patient's tumor antigens as vaccine components, which can more effectively guide the immune system to recognize and eliminate specific tumor cells, thereby avoiding the potential risks associated with non‐specific immune activation. Additionally, experimental results show that combining nECTs with an FMD can further enhance therapeutic efficacy, demonstrating the significant potential of this multimodal treatment approach in improving cancer therapy outcomes.

Additionally, the integration of cGAS‐STING‐targeted nanovaccines with extant tumor immunotherapies exhibits considerable potential for augmenting therapeutic efficacy. As a pivotal sensor of cytosolic DNA, the cGAS‐STING pathway occupies a central position in the activation of innate immune responses, which are capable of enhancing antitumor immunity through the augmentation of type I interferon and other pro‐inflammatory cytokine production. In combination therapy utilizing cGAS‐STING‐targeted nanovaccines and checkpoint inhibitors, the latter function by counteracting the immunosuppressive conditions within the tumor microenvironment, thus reinvigorating T‐cell functionality to effectively identify and eliminate cancer cells. This synergistic approach not only amplifies localized immune reactions but also elicits systemic anti‐tumor immune responses, markedly enhancing therapeutic efficacy and providing new prospects for patients with advanced or resistant tumors [65]. Moreover, integrating cGAS‐STING‐targeted nanovaccines with CAR‐T cell therapy represents a groundbreaking strategy in oncology. By leveraging nanotechnology to deliver cGAS‐STING agonists and augmenting the innate immune response, this method complements the action of genetically modified CAR‐T cells, which are engineered to precisely target and eradicate tumor cells [66]. This tandem approach is designed to intensify both local and systemic immune engagement, thereby optimizing tumor suppression and clinical outcomes.

## 4 Experimental Section/Methods

### 4.1 Materials

IPTG, FITC, indocyanine green (ICG), kanamycin, EDTA‐2Na, lysozyme, tris‐HCL, and glucose were purchased from Solarbio (Beijing, China). M9 minimal medium was acquired from Sigma (MO, USA). The procurement of glucose‐free Roswell Park Memorial Institute (RPMI) 1640 medium, standard RPMI 1640 medium, penicillin‐streptomycin, and phosphate‐buffered saline (PBS) was carried out through Beyotime (Shanghai, China). FBS was purchased from BIOIND (Beijing, China). Protein A/G Magnetic Beads were purchased from Biolinkedin (Shanghai, China). The LPS detection kit was purchased from BIOENDO (Xiamen, China). OVA, IL‐6, IL‐12p70, IFN‐γ, IFN‐β, TNF‐α, IgG, and IgG2c ELISA kits were obtained from LIANKE (Hangzhou, China). The C57BL/6 and BALB/C strains of mice were procured through SPF Biotechnology (Beijing, China).

### 4.2 Plasmid Construction and Transformation

Transformation of the *E. coli* strain BL21‐CodonPlus ‐RIL was performed using the pET28a‐SUMO expression plasmid that contained the m‐cGAS gene. Subsequent confirmation of the gene sequences was executed through DNA sequencing (ABIOCENTER, Beijing, China).

### 4.3 Preparation and Characterization of nECTs

To cultivate *E. coli*, the culture was incubated in M9 medium with agitation at 180 rpm and 37°C. Upon reaching an OD600 of approximately 0.6, the bacteria were further grown in the presence of 0.1 mM of IPTG under the same conditions but with increased shaking at 200 rpm for a duration of 20 h. Following this, *E. coli* cells were collected via centrifugation at 3000 g at 4°C for 10 min. Protoplast formation involved suspending the cells in a solution comprising 5 mM of EDTA‐2Na, 4 mg mL^−1^ of lysozyme, and 0.9% of glucose in 50 mM of Tris‐HCl buffer, which was then incubated at 37°C for half an hour to degrade the cell wall. Afterward, the protoplasts were isolated through centrifugation under identical parameters as before and underwent two wash cycles to eliminate residual outer membrane constituents. In the subsequent step, these protoplasts were combined with various tumor cells in 50 mM of Tris‐HCl buffer at a 1:1 feeding ratio based on total protein concentration. Ultrasound treatment at 35% intensity for 10 min facilitated the creation of nECTs. To remove larger debris, the suspension was initially spun down at 150 g at 4°C for 10 min. nECTs were then obtained by a second centrifugation at 18 000 g at the same temperature for 25 min. For nEs or nECs preparation, an equivalent quantity of *E. coli* was employed, and the procedure followed was consistent with that detailed above.

The dimensions alongside the zeta potentials of the nECTs were assessed utilizing the Zetasizer Nano ZS apparatus, manufactured by Malvern Instruments. For scrutinizing the structural characteristics of the nECTs, observations were carried out employing an HT7700 transmission electron microscope (Hitachi). To confirm the synthesis of 2′3′‐cGAMP, nECs were extensively fragmented to release the intracellular materials and analyzed by LC‐20AT HPLC (Shimadzu). A standard curve of 2′3′‐cGAMP was used to determine the concentration of 2′3′‐cGAMP in nECs or BnECTs. For protein analysis, 4T1, CT26, B16‐F10, and B16‐OVA tumor cells and nECTs were lysed with RIPA to extract proteins, and the quantification of concentrations was achieved through the utilization of a BCA assay kit. The protein content was appraised via SDS‐PAGE electrophoresis and Coomassie Brilliant Blue staining. Adhering to the guidelines provided by the supplier, LPS was isolated employing a phenol/chloroform mixture at a ratio of 5:1 and then measured using an LPS detection kit.

### 4.4 In Vitro Cell Culture

Cultivation of DC2.4 and RAW264.7 cells was carried out in RPMI 1640 and DMEM media, respectively, under conditions of 37°C within an environment saturated with 5% carbon dioxide and humidity. In the context of in vitro experiments involving FMD treatments, DC2.4 or RAW264.7 cells were maintained in glucose‐devoid RPMI 1640 or DMEM media, respectively, with modifications including a 1% fetal bovine serum addition and a 0.5 g/L of glucose supplementation.

### 4.5 Immunoblotting Assay

DC2.4 cells were plated in 6‐well dishes and incubated for 24 h in either standard or FMD media with diverse compositions. Post‐incubation, cells were harvested, rinsed thrice with PBS, and subjected to lysis utilizing RIPA buffer augmented with a 1% (v/v) protease inhibitor, specifically phenylmethylsulfonyl fluoride (PMSF). Protein quantification was performed using a BCA assay kit. Subsequently, 20 µg of total protein extracts were fractionated via SDS‐PAGE electrophoresis and electrotransferred on polyvinylidene difluoride membranes. Immunodetection was carried out on these membranes using the following primary antibodies: IGF‐2R (CST #14364, dilution 1:1000), IGF‐1R (CST #3027, dilution 1:1000), Tubulin (CST #3873, dilution 1:1000), CDK4 (Proteintech 11026, dilution 1:1000), GAPDH (Bioworld #AP0066, dilution 1:5000), LC3‐I/II (Proteintech #14600, dilution 1:1000), Ubiquitin (CST #3936, dilution 1:1000), p‐IRF3 (CST #29047, dilution 1:1000), cGAS (CST #31659, dilution 1:1000), TBK1 (CST #38066, dilution 1:1000), p‐TBK1 (CST #5483, dilution 1:1000), STING (CST #13647, dilution 1:1000), and p‐STING (CST #72971, dilution 1:1000). This protocol was also used for nECGs, B16‐F10 and BnECTs analysis. All the raw data of the western blot are given in Figure S40.

### 4.6 In Vitro Cell Imaging

Cells were harvested and subsequently plated within 35 mm dishes suitable for confocal microscopy analysis, at an initial concentration of 1 × 10^5^ cells per well. These were cultured in a conventional growth medium under conditions of 37°C for 24 h. Thereafter, these cells were exposed to either fresh conventional media or FMD for an additional period of 24 h. To evaluate cellular internalization, cells were co‐cultured with FITC‐labeled nEs, nECs, or BnECTs for timeframes of either 2 or 6 h. Post‐incubation, cells underwent staining with LysoTracker Red (50 nM) for 30 min to visualize lysosomal compartments. This was followed by immersion using 4% PFA at ambient temperature for 15 min. Nuclei were then highlighted through DAPI staining (300 nM) applied for 10 min under the same temperature conditions. Cells were rinsed thrice with PBS before visualization under a confocal microscope (Zeiss LSM880).

### 4.7 Immunofluorescence Staining

DC2.4 cells were collected and seeded into 35 mm dishes optimized for confocal microscopy analysis at a concentration of 1 × 10^5^ cells per well in the standard medium at 37°C for 24 h and switched to fresh standard or FMD medium for 1 day. The DC2.4 was incubated with nECTs for 24 h, and the fixation was achieved through the application of 4% PFA at ambient temperature for a duration of 10 min. Subsequent permeabilization of the cellular membranes was facilitated by immersing the cells in a PBS solution enhanced with 0.3% Triton X‐100 for 10 min. To minimize nonspecific binding, cells were subjected to a blocking step for 30 min.

Then, cells were incubated with appropriate primary antibodies cGAS (CST #31659, dilution 1:200), p‐STING (CST #72971, dilution 1:200), or p‐IRF3 (CST #29047, dilution 1:200) overnight at 4°C. Subsequently, DC2.4 cells were washed twice with PBS and incubated with the secondary antibodies Alexa Fluor 488 (ab150077, dilution 1:1000) or Alexa Fluor 594 (ab150080, dilution 1:1000) for 1 h at room temperature. Cell nuclei were stained with DAPI (300 nM) for 10 min in the room. Confocal imaging was conducted utilizing a confocal microscope (Zeiss LSM880).

Subsequently, the specimens were exposed to primary antibodies targeting cGAS (CST #31659), p‐STING (CST #72971), and p‐IRF3 (CST #29047), each diluted to 1:200, under refrigeration overnight. Post‐incubation, cells underwent a thorough rinsing process with PBS before being incubated with fluorescently labeled secondary antibodies, Alexa Fluor 488 (ab150077) and Alexa Fluor 594 (ab150080), both diluted to 1:1000, for an hour at room temperature. Nuclear staining was accomplished using a DAPI solution at a concentration of 300 nM for 10 min, under ambient conditions. High‐resolution imaging was conducted utilizing a confocal microscope (Zeiss LSM880), allowing for meticulous visualization and analysis of the cellular components and interactions.

For in vivo investigations, excised tumor specimens were promptly preserved in an optimal cutting temperature compound through snap‐freezing. Subsequent processing involved slicing the tumors into sections of 10 µm thickness. These sections underwent fixation in a 4% PFA solution for a 10‐min interval at room temperature, followed by rinsing in PBS on two occasions. To prepare the tissue for antibody penetration, sections were pre‐treated with sheep serum and a 0.3% Triton X‐100 solution in PBS for 15 min at room temperature. Antigen retrieval was then achieved by immersing the sections in Citrate Antigen Retrieval Solution for 1 h. This step was crucial for exposing antigens that would otherwise be masked within the tissue structure. Subsequently, the tumor sections were incubated with a primary antibody against CD8 (catalog #GB11068, used at a dilution of 1:1000) for immunofluorescence labeling. Post‐incubation, sections were thoroughly washed three times before the addition of an Alexa Fluor 488 labeled secondary antibody. Incubation with this secondary antibody took place at room temperature for an hour to ensure adequate binding. After completing the washing process, nuclear staining was performed using DAPI (at a concentration of 300 nM) for a duration of 10 min. Finally, all prepared sections were subjected to examination under a confocal microscope (Zeiss LSM880) to visualize the distribution and expression levels of the targeted antigens within the tumor microenvironment.

### 4.8 Flow Cytometric Analysis

Cells were exposed to diverse treatment formulations for durations of 2 or 6 h to evaluate their uptake kinetics. Post‐treatment, the cells underwent three rounds of PBS cleansing to remove any unbound substances. Subsequently, they were harvested, immobilized using a fixation buffer, and subjected to analysis employing a flow cytometer (BD Accuri C6) to quantify internalization. For in vivo flow cytometry examination, tumors and lymph nodes were surgically retrieved and manually dissociated to generate single‐cell suspensions. Within these suspensions, erythrocytes were selectively eliminated by immersion in red blood cell lysis buffer. Before antibody staining, the cells were blocked with anti‐CD16/CD32 antibodies to avoid non‐specific binding. Thereafter, the samples were labeled with fluorescence‐tagged antibodies against mouse antigens for specific cell population identification: AF700‐anti‐CD45 (Biolegend 103108, dilution 1:100), FITC‐anti‐CD3 (Biolegend 100204, dilution 1:100), APC‐Cyanine 7‐anti‐CD8 (Biolegend 100713, dilution 1:100), PE‐anti‐CD4 (Biolegend 100512, dilution 1:100), Brilliant Violet 421‐anti‐IFN‐γ (Biolegend 505829, dilution 1:100), APC‐anti‐TNF‐α (Biolegend 506308, dilution 1:100), APC‐anti‐F4/80 (Biolegend 123115, dilution 1:100), Brilliant Violet 605‐anti‐CD11b (Biolegend 101257, dilution 1:100), PE‐anti‐CD206 (Biolegend 141706, dilution 1:100), APC‐anti‐NK1.1 (Biolegend 480050, dilution 1:100), and FITC‐anti‐IA/IE (Biolegend 107605, dilution 1:100).

To conduct intracellular staining, cells underwent initial activation utilizing a cell activation cocktail inclusive of Brefeldin A, which was administered throughout 6 h. Post‐activation, cells were subjected to fixation with a fixation buffer, followed by permeabilization facilitated by an intracellular staining permeabilization wash buffer. After these preparatory steps, cells were subjected to a flow cytometer (BD Biosciences LSRFortessa). The acquired data were meticulously analyzed through the application of FlowJo software. An illustrative representation of the gating strategy employed during the flow cytometry analyses can be found in Figures S41 or S42.

For in vitro studies, BMDCs were specifically harvested from the tibias and femurs of juvenile C57BL/6 mice aged approximately 6 weeks. The harvested cells were subsequently cultured in a growth medium, and cytokines, including 10 ng mL^−1^ of GM‐CSF (BioLegend) and 10 ng mL^−1^ of IL‐4. Cells were washed on Days 1, 3, and 5 and re‐plated on Day 7 for further studies. To determine the maturation of BMDCs, BMDCs were incubated with 2′3′‐cGAMP, nEs, nECs, or BnECTs for 24 h and collected in FACS buffer containing 1% of FBS. The BMDCs were blocked with anti‐CD16/32 antibodies at ambient temperature for 30 min and then labeled with a panel of fluorescently conjugated antibodies targeting specific surface markers, including CD11c (Biolegend 117308, dilution 1:100), CD86 (Biolegend 105012, dilution 1:100), CD80 (Biolegend 104726, dilution 1:100), CD40 (Biolegend 124622, dilution 1:100), and MHC‐I (Biolegend 114618, dilution 1:100) for 30 min before analysis by a flow cytometer (BD Accuri C6).

### 4.9 Detections of Cytokines

The secretions of IL‐6, IL‐12p70, TNF‐α, and IFN‐β in DCs cultured medium were checked by ELISA analyses following the manufacturer's instructions. For in vivo detections, tumor tissue from saline, 2′3′‐cGAMP, nEs, nECs, BnECTs, OnECTs, 4nECTs, or CnECTs groups was first detached into single cells. After being incubated in red blood cell lysis buffer at ambient temperature for 10 min, the cells were collected via centrifugation at 150 g at 4°C for 5 min, and they were washed with PBS and subjected to lysis through resuspension in RIPA buffer containing 1% PMSF. Finally, the levels of IgG, IgG2c, IL‐6, IL‐12p70, TNF‐α, IFN‐β, and IFN‐γ were evaluated via ELISA following the manufacturer's instructions.

### 4.10 In Vivo Anti‐Tumor Studies

To obtain tumor models in C57BL/6 or BALB/C mice, 5 × 10^5^ viable 4T1, CT26, B16‐F10, or B16‐OVA tumor cells were suspended in a 1:1 of PBS: Matrigel mixture and injected subcutaneously into the flanks of 6–8‐week‐old female C57BL/6 or BALB/C mice. Once tumor volumes reached approximately 100 mm^3^, treatment regimens included administration of 100 µL of saline, free 2′3′‐cGAMP (0.2 mg kg^−1^), naked extracellular vesicles (nEs; 4.5 mg kg^−1^ protein), naked extracellular carriers (nECs; 4.5 mg kg^−1^ protein containing 4 µg 2′3′‐cGAMP), BnECTs, OnECTs, 4nECTs, and CnECTs (all at 4.5 mg kg^−1^ protein containing 4 µg 2′3′‐cGAMP). FMD interventions were scheduled on days 6–8, 11–13, and 16–18. Each FMD cycle consisted of a single day of a diet reduced by 50% in calories, followed by 2 days of a diet reduced by 90% in calories. Tumor volume measurements were calculated using the formula: *V* = *L* × *S* ^2^ / 2, where *L* represents the longest tumor diameter and *S* the shortest diameter, ascertained via caliper measurements. Daily monitoring of body weight was conducted throughout the study. Upon conclusion of the experimental period, mice were humanely euthanized following institutional animal care guidelines. Tumors, along with vital organs, were meticulously excised, weighed, and preserved for subsequent histopathological analysis.

### 4.11 Lymph Node Imaging

On the second day of the FMD cycle, C57BL/6 mice were subcutaneously injected with the ICG‐labeled BnECTs or free ICG. The mice were sacrificed 24 h after the injection, and the lymph nodes were isolated, which were detected using an in vivo optical imaging system (IVIS Spectrum, PerkinElmer). The fluorescence intensity of lymph nodes and organs was analyzed with Living Image 4.1 software.

In the progression of the FMD on its second day, mice of the C57BL/6 lineage were administered a subcutaneous dose at the tail origin, featuring either BnECTs tagged with ICG or just ICG as a control. After enduring a duration of 24 h post‐administration, the subjects were humanely terminated, and their lymph nodes were carefully retrieved for a trio of specimens. Subsequently, the gathered lymph nodes underwent scrutiny employing an innovative in vivo optical imaging mechanism, notably the IVIS Spectrum model from PerkinElmer, which facilitated the detection and quantification of the fluorescent emissions from the ICG‐marked components within these nodes and additional organs. This process involved the employment of Living Image 4.1 software to meticulously analyze the fluorescence intensities.

### 4.12 Immunohistochemistry Staining

The tissue samples underwent fixation via immersion in a solution containing 4% PFA for a duration of 10 min under ambient conditions. Subsequently, they were rinsed in PBS on two occasions. After the blocking process, the sections were exposed to primary antibodies targeting p‐STING (CST #72971, dilution 1:200) or p‐IRF3 (CST #29047, dilution 1:200) antibodies, with overnight incubation at 4°C. Following this, the sections were subjected to thorough washing with a wash buffer on no less than three separate occasions. They were then overlaid with SignalStain Boost IHC Detection Reagent and maintained at room temperature for 1 h. Post‐incubation, each sample received a 100 µL dose of SignalStain DAB solution and was allowed to react for 5 min. The sections were subsequently submerged in deionized water across three cycles. For quantifying the immunohistochemical expression of p‐STING and p‐IRF3 within the tumor sections, assessments were made considering both the extent and the intensity of the staining. Slides of five tumors for each group were calculated using Image‐Pro Plus 6.0.

### 4.13 In Vivo Safety Evaluation

Mice were humanely euthanized via exposure to CO_2_, and major organs were isolated and promptly immersed in 4% PFA for staining with H&E to evaluate the biosafety of FMD treatment or nECTs. The collected blood was further analyzed for routine blood tests and biochemistry assays.

### 4.14 Statistical Analysis

In the current study, data representation primarily adopts the format of mean ± standard deviation (s.d.), unless alternative specifications are explicitly noted within individual figure legends. For scenarios involving comparative analyses, a one‐way analysis of variance (ANOVA) serves as the statistical tool of choice. All statistical operations are executed utilizing GraphPad Prism 8. Statistical significance is established at a *p*‐value less than 0.05.

## Author Contributions

**Wenping Huang**: conceptualization, methodology, investigation, visualization, writing – original draft. **Guoliang Cao**: methodology, investigation. **Mixiao Tan**: methodology, investigation. **Fuhao Jia**: investigation. **Jie Zhang**: investigation. **Wen Su**: investigation. **Yue Yin**: conceptualization, methodology, investigation, visualization, funding acquisition, project administration, supervision, writing – review and editing. **Hai Wang**: conceptualization, methodology, visualization, funding acquisition, project administration, supervision, writing – review and editing.

## Conflicts of Interest

The authors declare no conflicts of interest.

## Supporting information




**Supporting Information file 1**: exp270077‐sup‐0001‐SuppMat.docx

## Data Availability

The data supporting this study's findings are available from the corresponding author upon reasonable request.
