# Supplementary material for: Personalized Cancer Immunotherapy Boosted by cGAS‐STING‐Targeted Nanovaccines in Combination With Nutrient Modulation
Source: Exploration (Beijing). 2025 Aug 22;5(6):20240183. doi: 10.1002/EXP.20240183 (PMC12752548; doi:10.1002/EXP.20240183)
Supplement: Supplementary file 1 — Supporting Information file 1: exp270077‐sup‐0001‐SuppMat.docx [file EXP2-5-20240183-s001.docx]

Supporting Information

Personalized Cancer Immunotherapy Boosted by cGAS-STING-Targeted Nanovaccines in Combination with Nutrient Modulation

Wenping Huang, Guoliang Cao, Mixiao Tan, Fuhao Jia, Jie Zhang, Wen Su, Yue Yin*, Hai Wang*


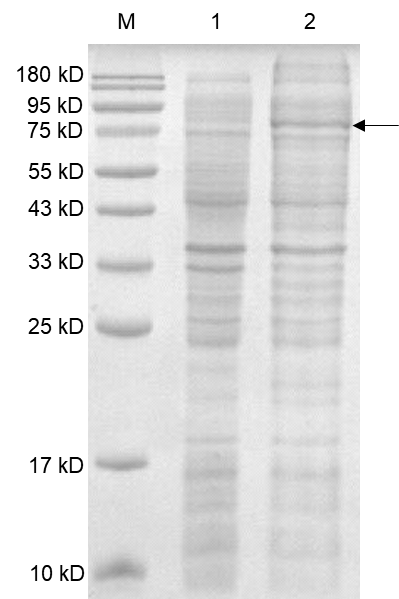


**Figure S1.** SDS-PAGE gel staining of proteins in *E. coli*. M, protein marker. Lane 1: protein lysates from *E. coli* without genetic engineering. Lane 2: protein lysates from 2′3′-cGAMP@*E. coli* expressing m-cGAS. The arrow indicates the band associated with the m-cGAS protein.


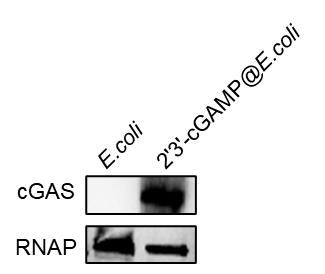


**Figure S2.** Western blotting analysis of cGAS in 2′3′-cGAMP@*E. coli*.

**
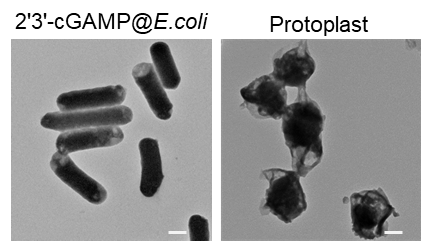
**

**Figure S3.** TEM images of the 2'3'-cGAMP@*E.coli*, protoplasts. Scale bars: 500 nm.

**
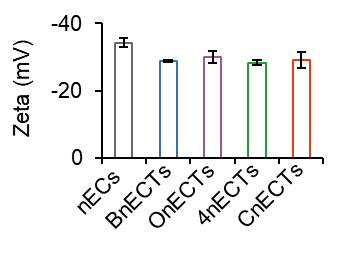
**

**Figure S4.** Zeta potential of nECs, BnECTs, OnECTs, 4nECTs, or CnECTs determined by DLS at room temperature. Error bars represent ± s.d. (n = 3).

**
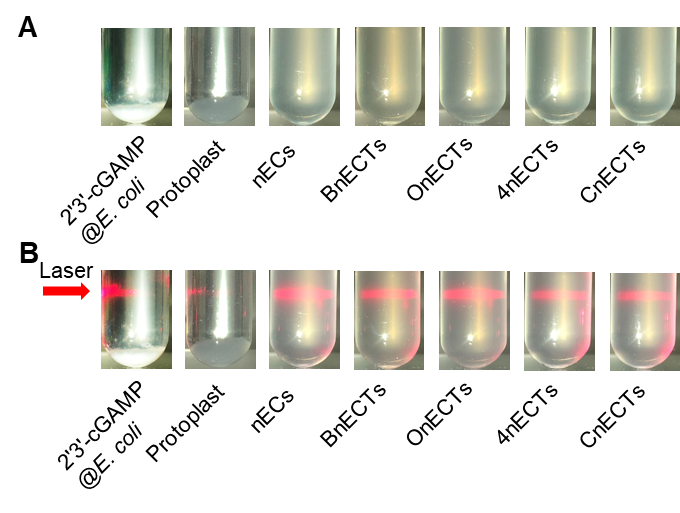
**

**Figure S5.** Stability of 2'3'-cGAMP@*E. coil,* protoplast, and nECTs. (A) A typical photograph of 2'3'-cGAMP@*E. coil*, protoplast, nECs, BnECTs, OnECTs, 4nECTs, or CnECTs in PBS solution. The nECTs could stably disperse in PBS solution while 2'3'-cGAMP@*E. coil* and protoplasts aggregated at the bottom of the tube. (B) Photograph of solutions after shining a red laser beam (arrow). The light track could only be observed in nECs, BnECTs, OnECTs, 4nECTs, or CnECTs due to the Tyndall effect.

**
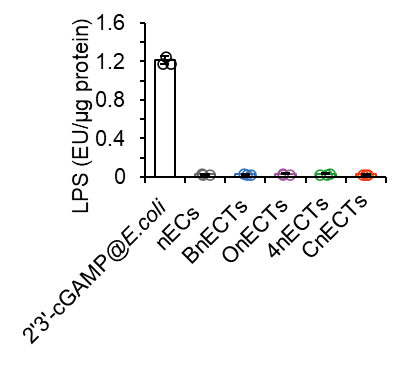
**

**Figure S6.** The concentrations of LPS in 2'3'-cGAMP@*E. coil*, nECs, BnECTs, OnECTs, 4nECTs, and CnECTs were determined by ToxinSensor™ Chromogenic LAL Endotoxin Assay Kit. Error bars represent ± s.d. (n = 3).


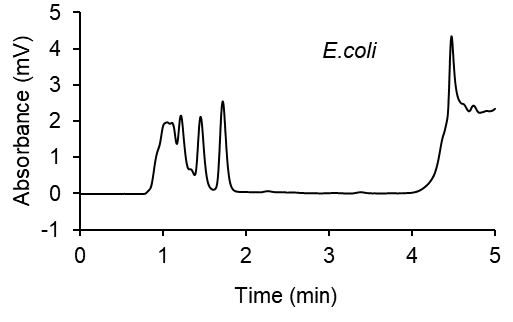


**Figure S7.** HPLC analysis for ECs of normal *E. coli*.

**
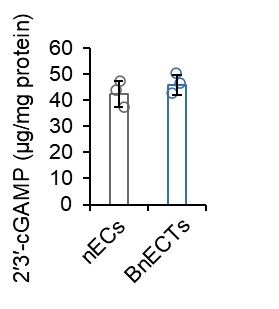
**

**Figure S8.** The concentrations of 2′3′-cGAMP in nECs and BnECTs. Error bars represent ± s.d. (n = 3).


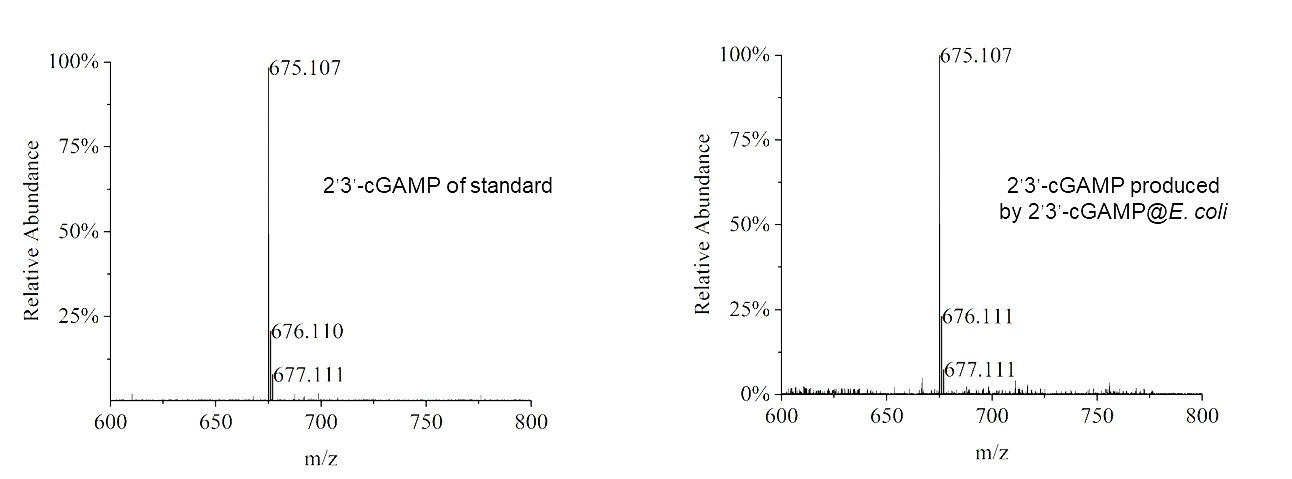


**Figure S9.** Mass spectrometry analysis for 2′3′-cGAMP produced by 2′3′-cGAMP @*E. coli* and the standard 2′3′-cGAMP.


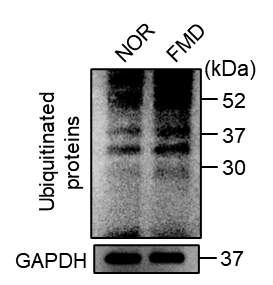


**Figure S10.** Western blotting analysis of ubiquitinated protein in normal (NOR) or FMD medium cultured DCs.


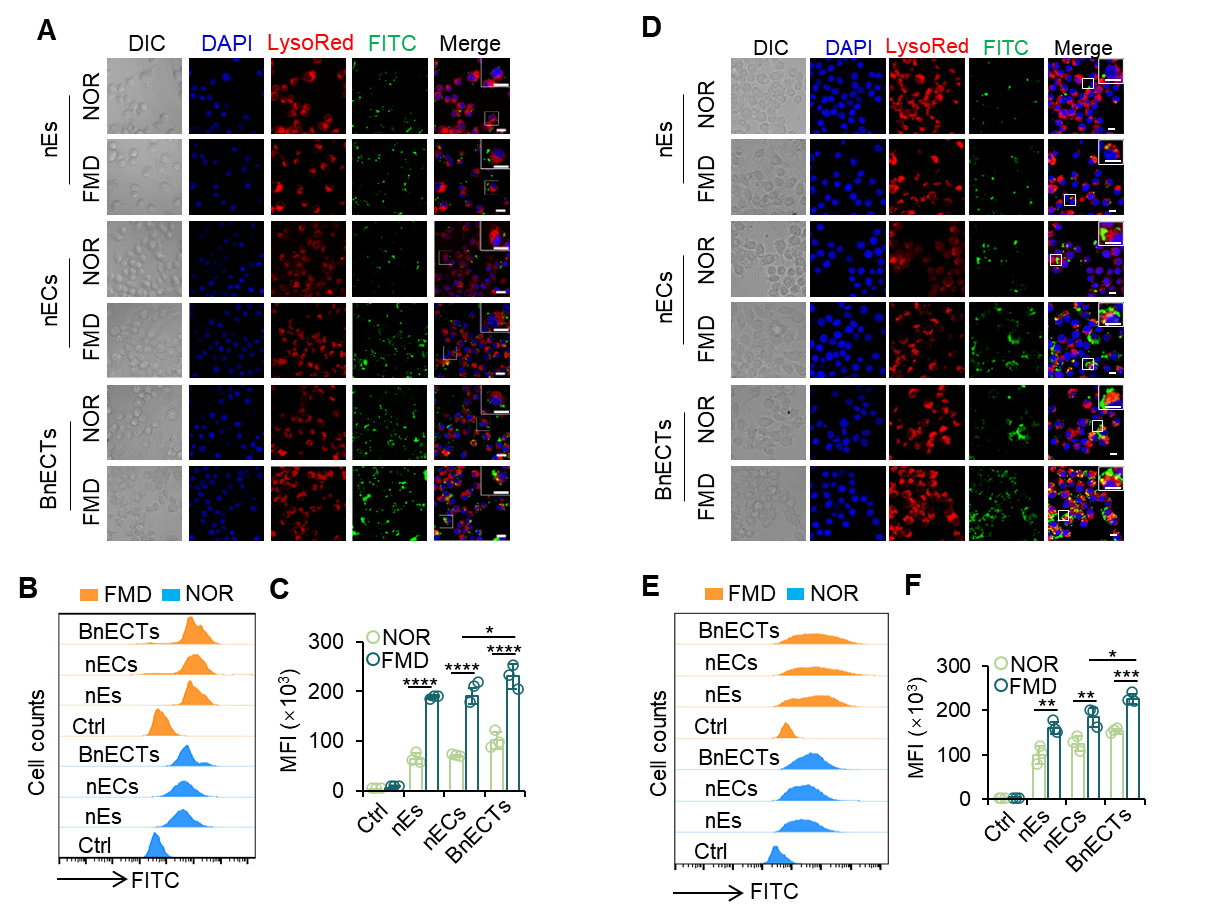


**Figure S11.** FMD treatment enhanced the cellular uptake of nECTs in DCs and RAW264.7. (A) Representation confocal images of DC2.4 cells incubated with FITC-labelled nEs, nECs, or BnECTs for 2 h. The nuclei and endo/lysosomes were stained with DAPI and LysoTracker Red (LysoRed), respectively. Scale bar, 10 μm. (B) Flow cytometry data and (C) mean fluorescence intensity (MFI) in DCs treated with FITC-labelled nEs, nECs, or BnECTs for 2 h. Error bars represent ± s.d. (n = 3). (D) Representation confocal images of RAW264.7 cells incubated with FITC-labelled nEs, nECs, or BnECTs for 2 h. The nuclei and endo/lysosomes were stained with DAPI and LysoTracker Red (LysoRed), respectively. Scale bar, 10 μm. (E) Flow cytometry data and (F) mean fluorescence intensity (MFI) in RAW264.7 treated with FITC-labelled nEs, nECs, or BnECTs for 2 h. Error bars represent ± s.d. (n = 3). Statistical significance was assessed by one-way ANOVA with turkey test. **p* < 0.05, ***p* < 0.01, ****p* < 0.001, *****p* < 0.0001.


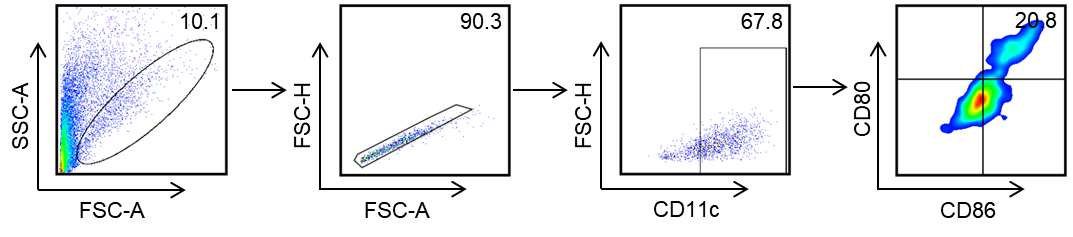


**Figure S12.** Flow cytometry gating strategy for the analysis of Figure 3F.


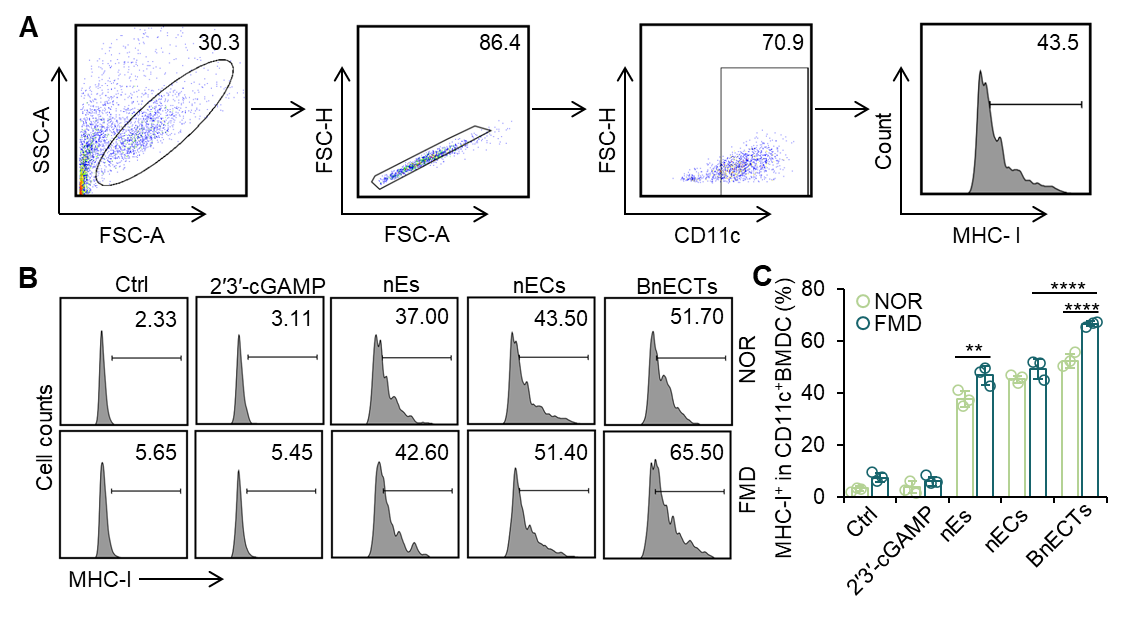


**Figure S13.** (A) Flow cytometry gating strategy, (B) flow cytometry data, and (C) quantitative analysis of MHC-I^+^ BMDCs with various treatments for 24 h. Statistical significance was assessed by one-way ANOVA with turkey test. ***p* < 0.01, *****p* < 0.0001.


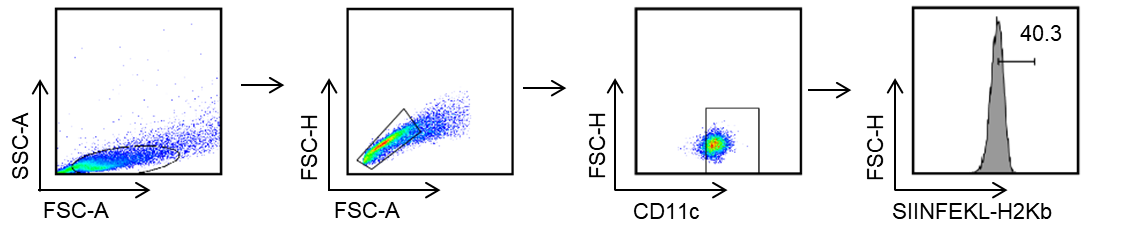


**Figure S14.** Flow cytometry gating strategy for the analysis of Figure 3K.


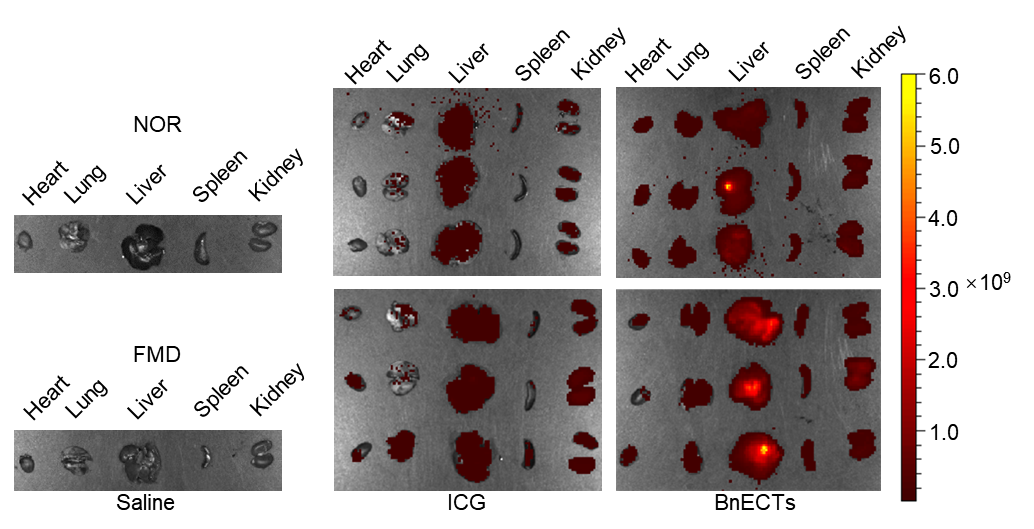


**Figure S15.** *Ex vivo* IVIS imaging of major organs. Free ICG or ICG-labelled BnECTs were subcutaneously injected into mice, and major organs were harvested at 24 h post-injection and imaged with an IVIS imaging system.


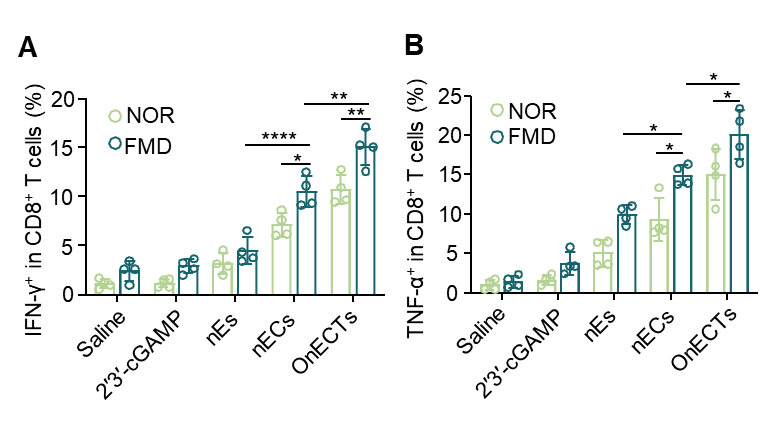


**Figure S16.** Quantitative analysis of (A) IFN-γ-positive cells and (B) TNF-α-positive cells in CD3^+^CD8^+^ T cells in lymph nodes at Day 20 post-immunization of C57BL/6 mice. Error bars represent ± s.d. (n = 4). Statistical significance was assessed by one-way ANOVA with turkey test. **p* < 0.05, ***p* < 0.01, *****p* < 0.0001.


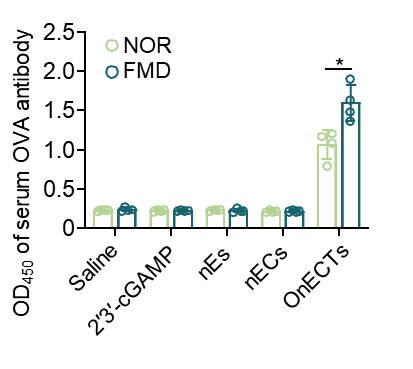


**Figure S17.** Serum OVA IgG in the C57BL/6 with various treatments. Error bars represent ± s.d. (n = 4). Statistical significance was assessed by one-way ANOVA with turkey test. **p* < 0.05.


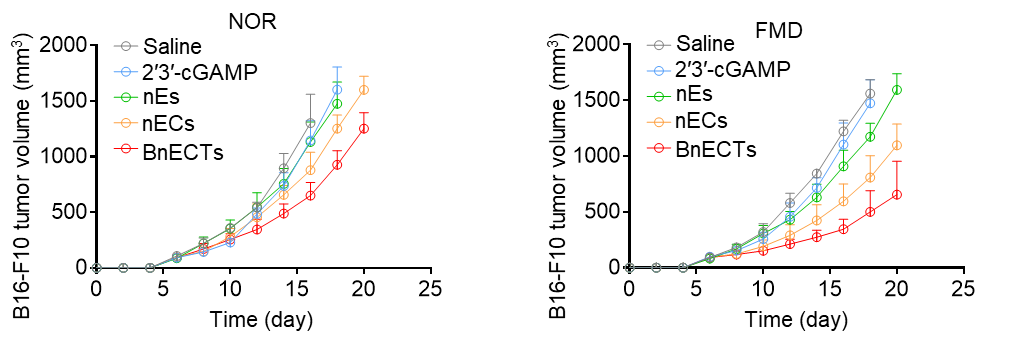


**Figure S18.** Tumor growth curves of B16-F10 tumors with various treatments. Error bars represent ± s.d. (n = 6).


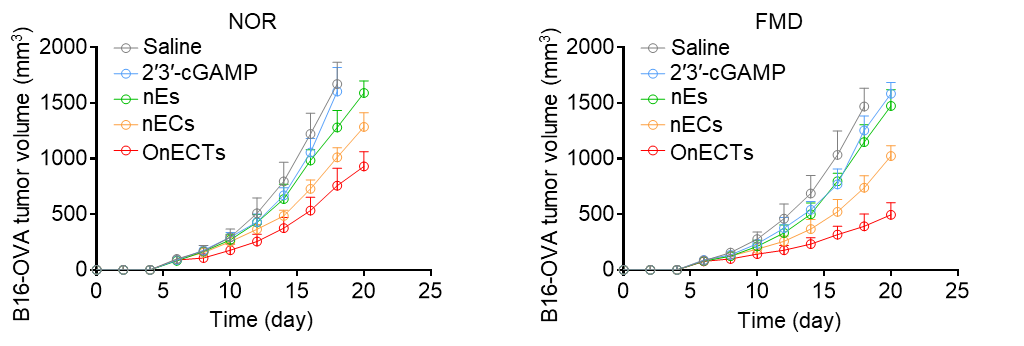


**Figure S19.** Tumor growth curves of B16-OVA tumors with various treatments. Error bars represent ± s.d. (n = 6).


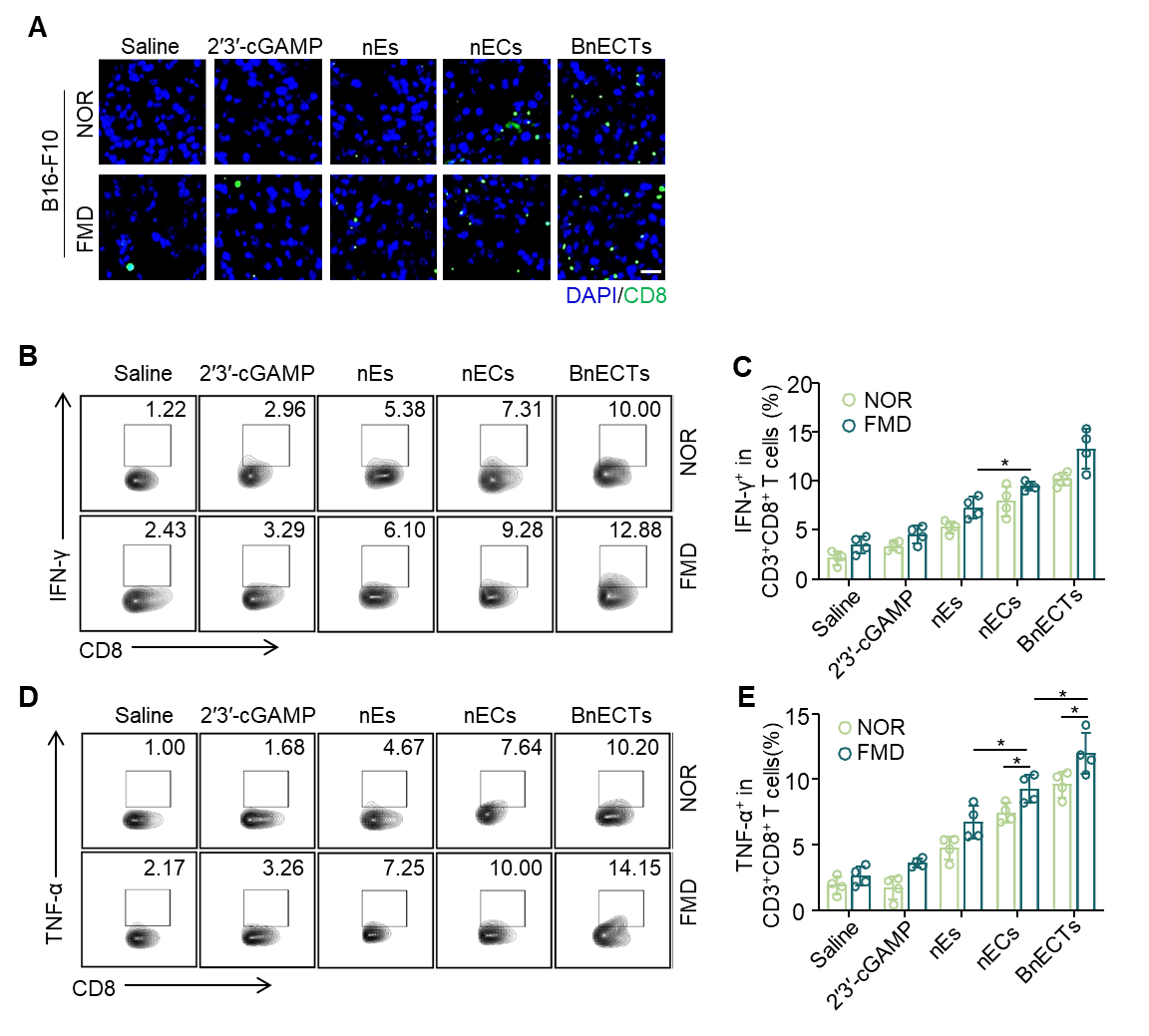


**Figure S20.** (A) Confocal images of infiltrated CD8^+^ T cells in the B16-F10 tumors. Scale bar, 50 μm. (B) Flow cytometry data and (C) quantitative analysis of IFN-γ-positive cells in CD3^+^CD8^+^ T cells in B16-F10 tumors. Error bars represent ± s.d. (n = 4). (D) Flow cytometry data and (E) quantitative analysis of TNF-α-positive cells in CD3^+^CD8^+^ T cells in B16-F10 tumors. Error bars represent ± s.d. (n = 4). Statistical significance was assessed by one-way ANOVA with turkey test. **p* < 0.05.


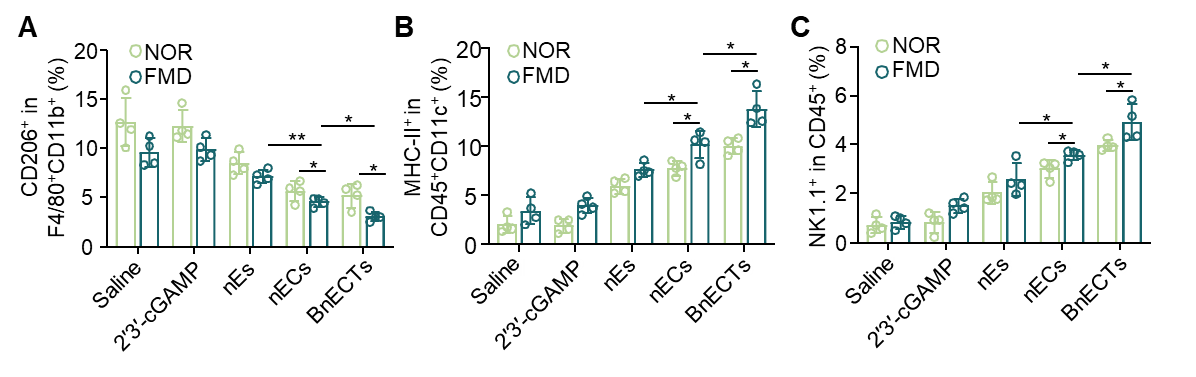


**Figure S21.** Quantitative analysis of (A) CD206-positive macrophages, (B) MHC-II-positive DCs, and (C) NK1.1 cells in B16-F10 tumors. Error bars represent ± s.d. (n = 4). Statistical significance was assessed by one-way ANOVA with turkey test. **p* < 0.05, ***p* < 0.01.


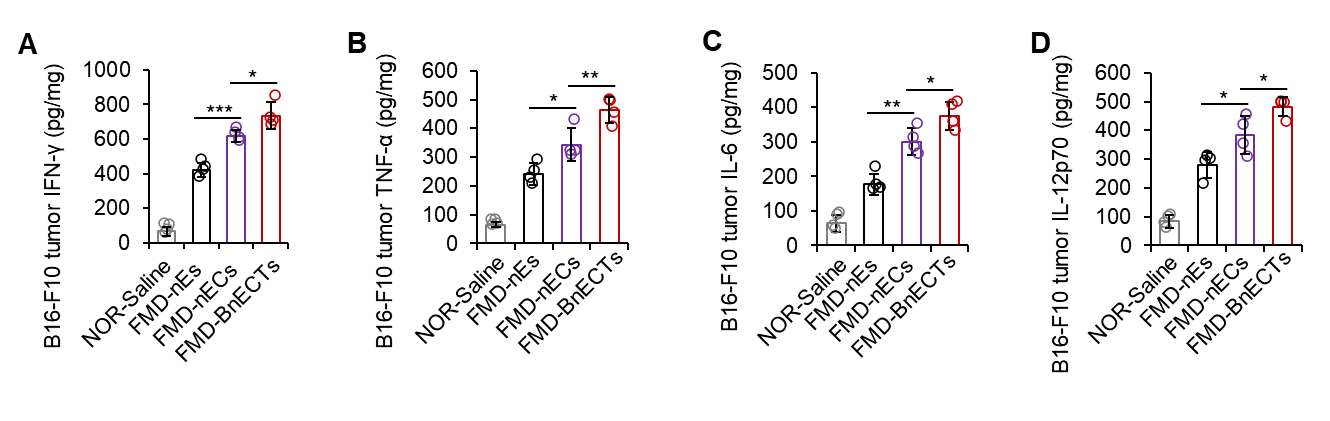


**Figure S22.** Quantitative analysis of (A) IFN-γ, (B) TNF-α, (C) IL-6, and (D) IL-12p70 in the B16-F10 tumors measured by ELISA kits. Error bars represent ± s.d. (n = 4). Statistical significance was assessed by one-way ANOVA with turkey test. **p* < 0.05, ***p* < 0.01, ****p* < 0.001.


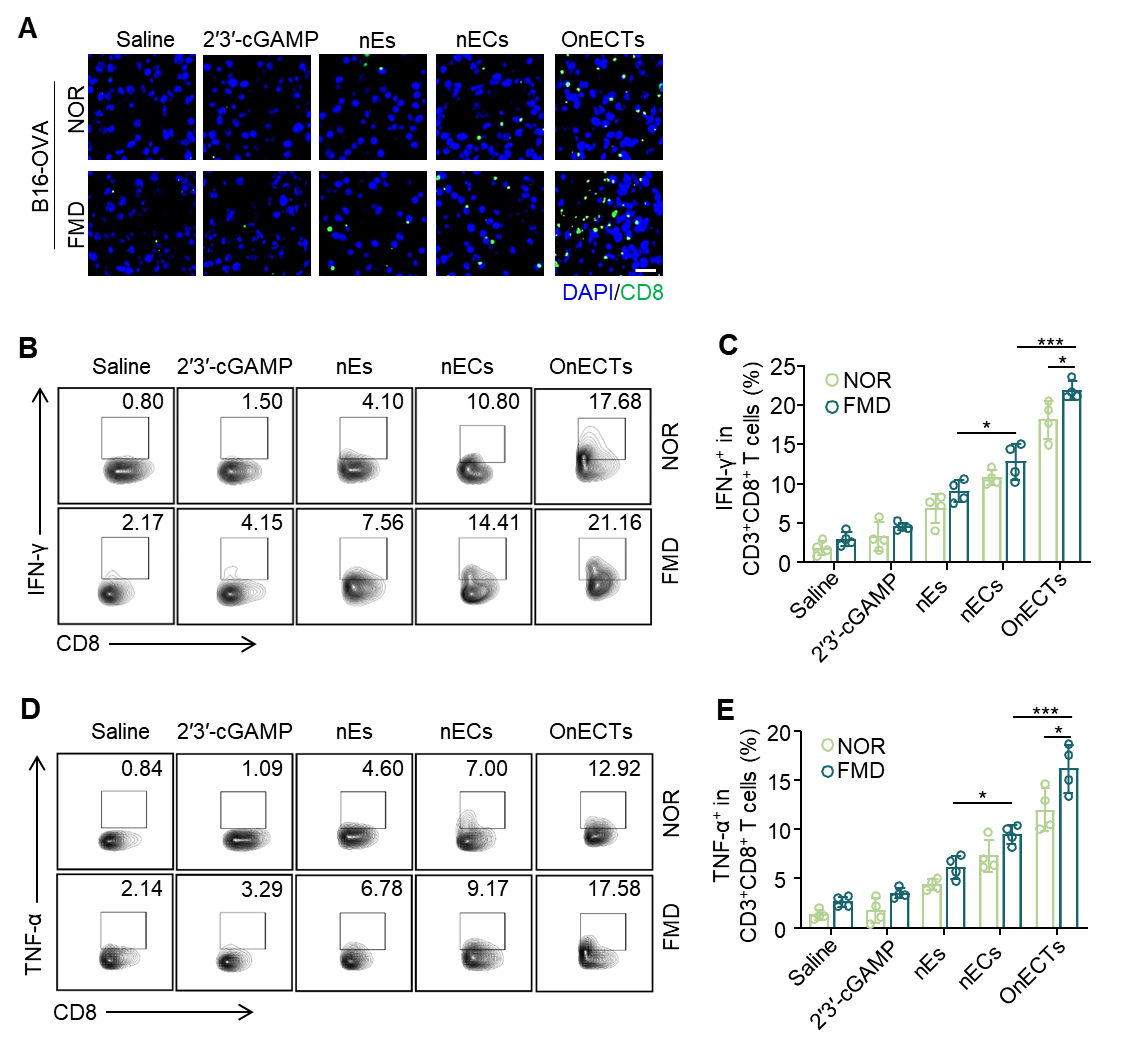


**Figure S23.** (A) Confocal images of infiltrated CD8^+^ T cells in the B16-OVA tumors. Scale bar, 50 μm. (B) Flow cytometry data and (C) quantitative analysis of IFN-γ-positive cells in CD3^+^CD8^+^ T cells in B16-OVA tumors. Error bars represent ± s.d. (n = 4). (D) Flow cytometry data and (E) quantitative analysis of TNF-α-positive cells in CD3^+^CD8^+^ T cells in B16-OVA tumors. Error bars represent ± s.d. (n = 4). Statistical significance was assessed by one-way ANOVA with turkey test. **p* < 0.05, ****p* < 0.001.


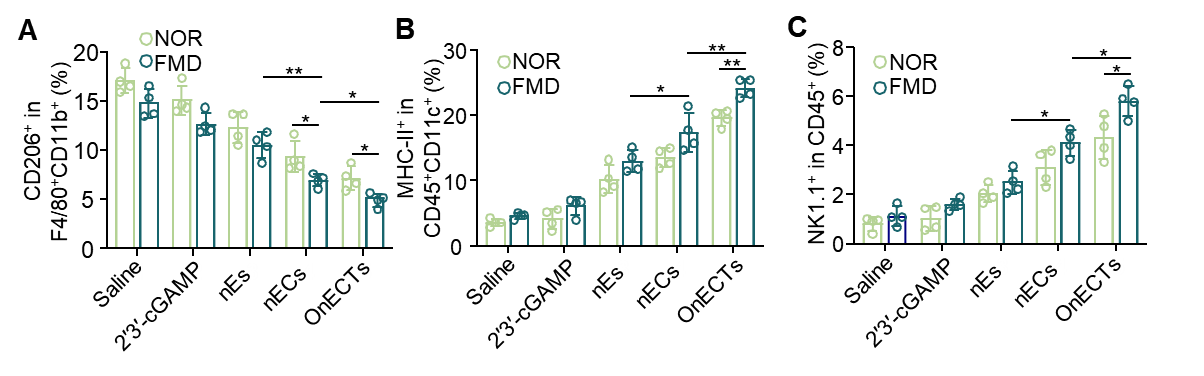


**Figure S24.** Quantitative analysis of (A) CD206-positive macrophages, (B) MHC-II-positive DCs, and (C) NK1.1 cells in B16-OVA tumors. Error bars represent ± s.d. (n = 4). Statistical significance was assessed by one-way ANOVA with turkey test. **p* < 0.05, ***p* < 0.01.


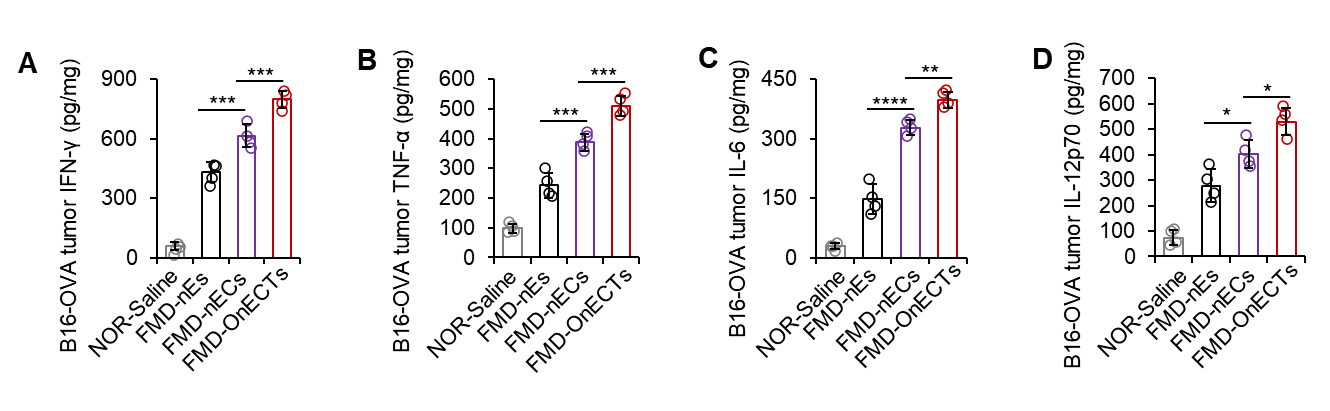


**Figure S25.** Quantitative analysis of (A) IFN-γ, (B) TNF-α, (C) IL-6, and (D) IL-12p70 in the B16-OVA tumors measured by ELISA kits. Error bars represent ± s.d. (n = 4). Statistical significance was assessed by one-way ANOVA with turkey test. **p* < 0.05, ***p* < 0.01, ****p* < 0.001, *****p* < 0.0001.


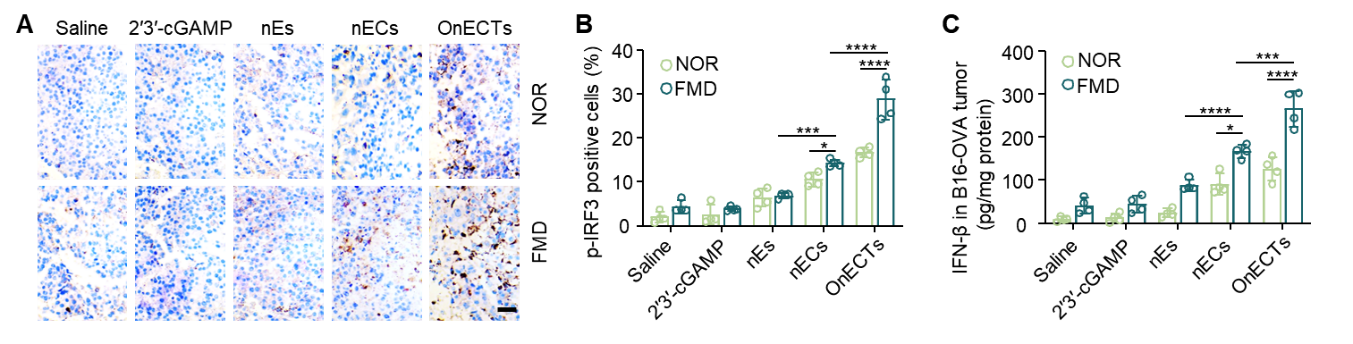


**Figure S26.** (A) Immunohistochemical images of p-IRF3 expressed cells (brown) in B16-OVA tumor tissues. Scale bar, 50 μm. (B) Quantitative analysis of p-IRF3 positive cells in the B16-OVA tumors. Error bars represent ± s.d. (n = 4). (C) Quantitative analysis of IFN-β in the B16-OVA tumors. Error bars represent ± s.d. (n = 4). Statistical significance was assessed by One-way ANOVA with turkey test. **p* < 0.05, ****p* < 0.001, *****p* < 0.0001.


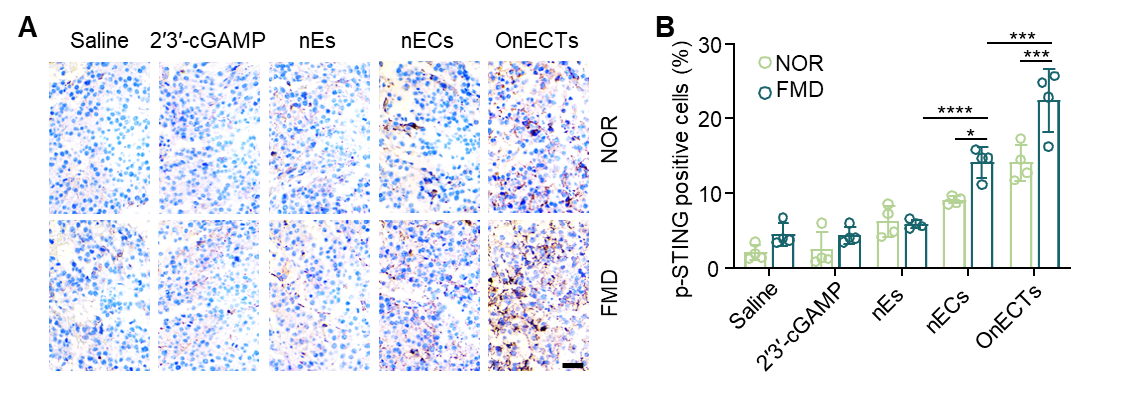


**Figure S27**. (A) Immunohistochemical images of p-STING expressed cells (brown) in B16-OVA tumor tissues. Scale bar, 50 μm. (B) Quantitative analysis of p-STING positive cells in the B16-OVA tumor tissues. Error bars represent ± s.d. (n = 4). Statistical significance was assessed by one-way ANOVA with tukey test. **p* < 0.05, ****p* < 0.001, *****p* < 0.0001.

**
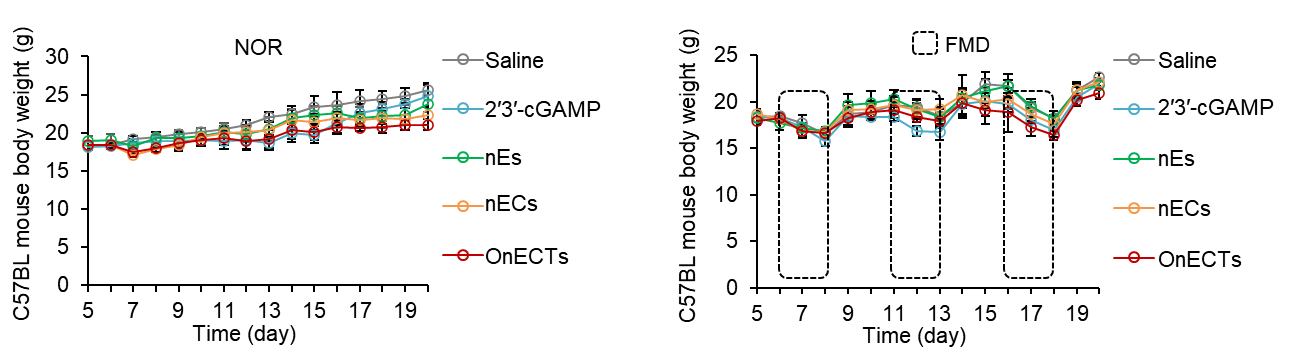
**

**Figure S28.** Body weights curve of C57BL6 mice bearing B16-OVA tumors. Error bars represent ± s.d. (n = 6).


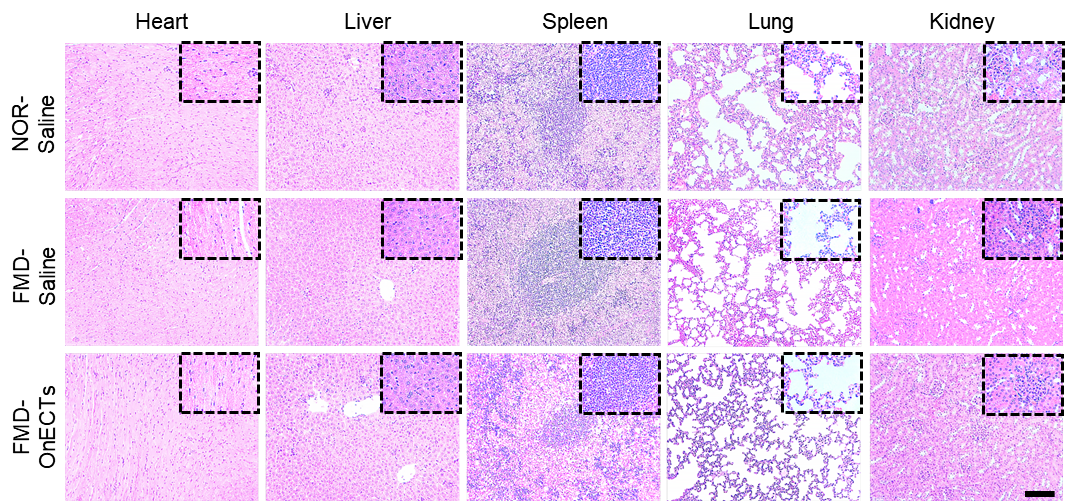


**Figure S29**. H&E staining of major organs excised from C57BL/6 mice 20 days after treatments of OnECTs under FMD condition. No noticeable signs of organ damage appeared in all major organs of mice.


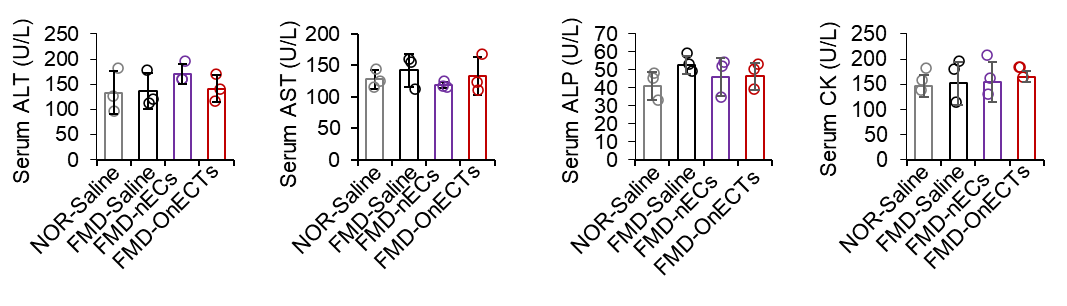


**Figure S30**. Quantitative analysis of the ALT, AST, ALP, and CK test of mice treated with OnECTs under FMD condition. Error bars represent ± s.d. (n = 3).


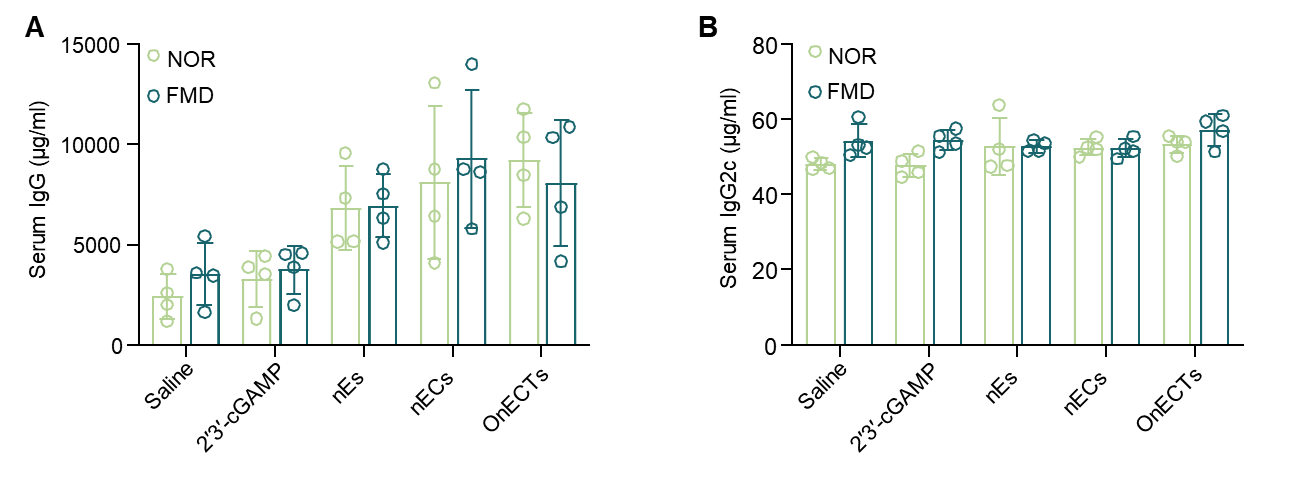


**Figure S31**. Quantitative analysis of (A) IgG and (B) IgG2c were measured in the serum from C57BL/6 mice at the end of the experiment. Error bars represent ± s.d. (n = 4).


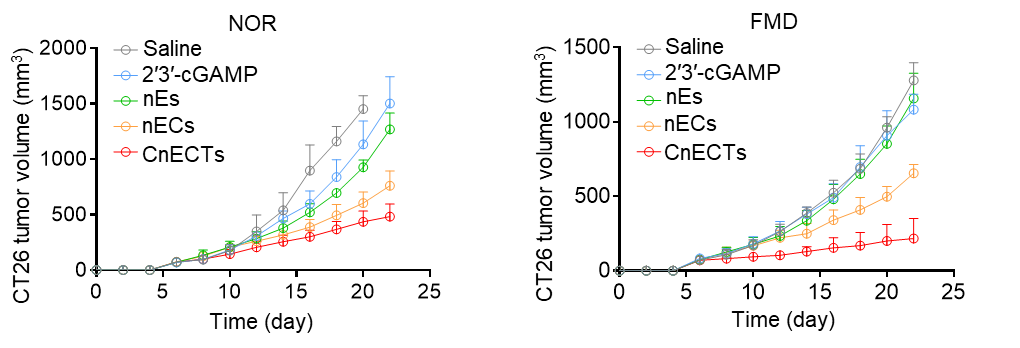


**Figure S32.** Tumor growth curves of CT26 tumors with various treatments. Error bars represent ± s.d. (n = 6).


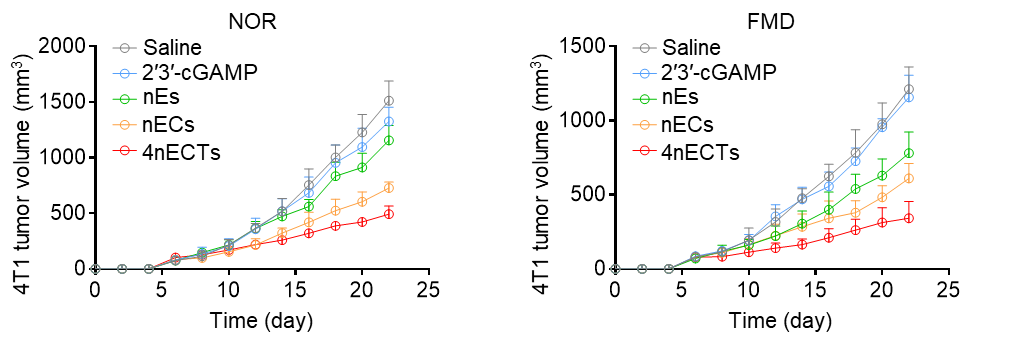


**Figure S33.** Tumor growth curves of 4T1 tumors with various treatments. Error bars represent ± s.d. (n = 6).

**
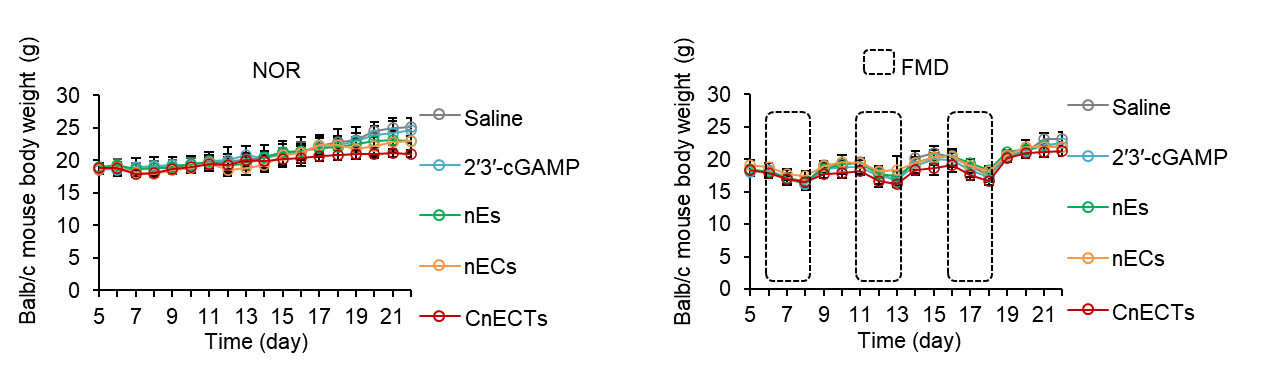
**

**Figure S34.** Body weights curve of Balb/c mice bearing CT26 tumors. Error bars represent ± s.d. (n = 6).


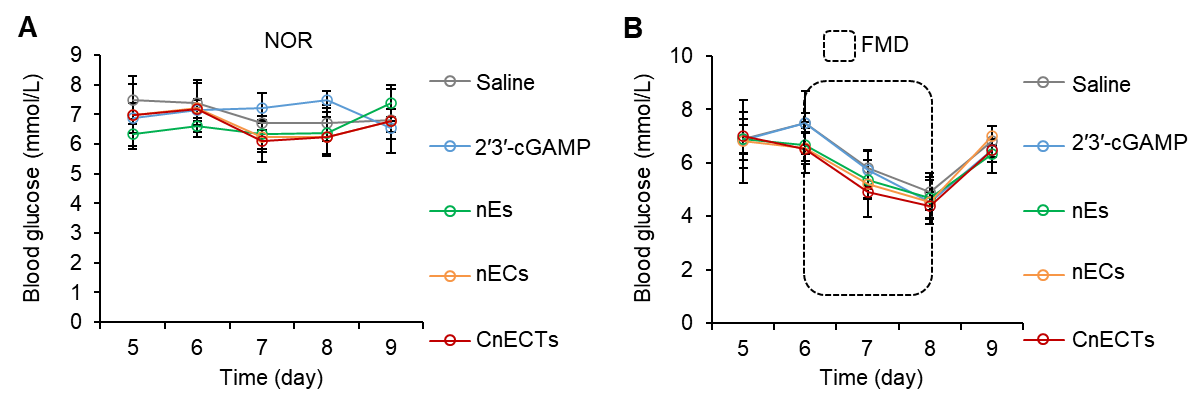


**Figure S35.** Blood glucose was measured during (A) NOR or (B) FMD treatments. Error bars represent ± s.d. (n = 6).


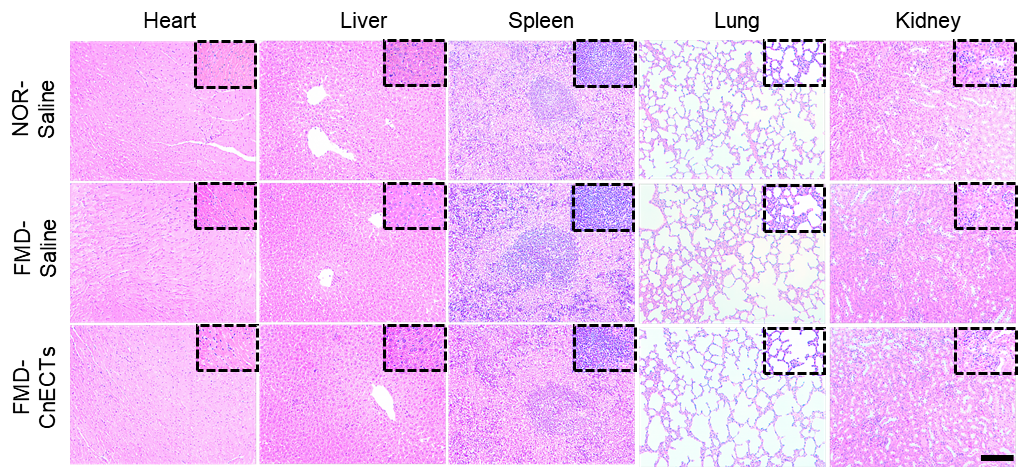


**Figure S36.** H&E staining of major organs excised from BALB/C mice 22 days after treatments of CnECTs under FMD condition. No noticeable signs of organ damage appeared in all major organs of mice.


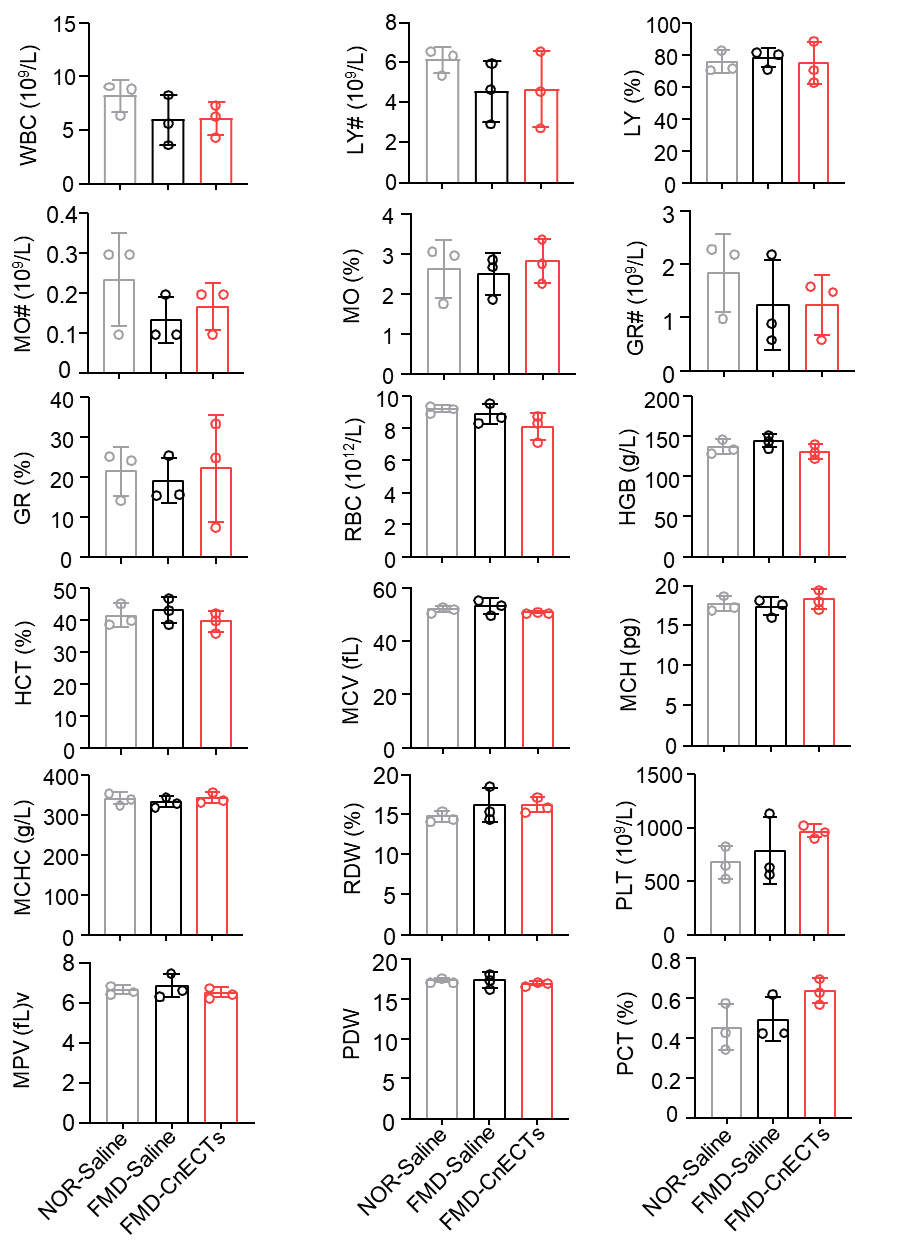


**Figure S37.** The blood samples were collected on Day 22 for further analysis. White blood cell (WBC), lymphocyte (LY), monocytes (MON), granulocyte (GR), red blood cells (RBC), hemoglobin (HGB), hematocrit (HCT), mean corpuscular volume (MCV), mean corpuscular hemoglobin (MCH), mean corpuscular hemoglobin concentration (MCHC), red cell distribution width (RDW), platelet count (PLT), mean platelet volume (MPV), platelet distribution width (PDW) and platelet crit (PCT) were analyzed. Compared to the normal group, no statistical difference was observed in FMD or CnECTs treated mice. Error bars represent ± s.d. (n = 3).


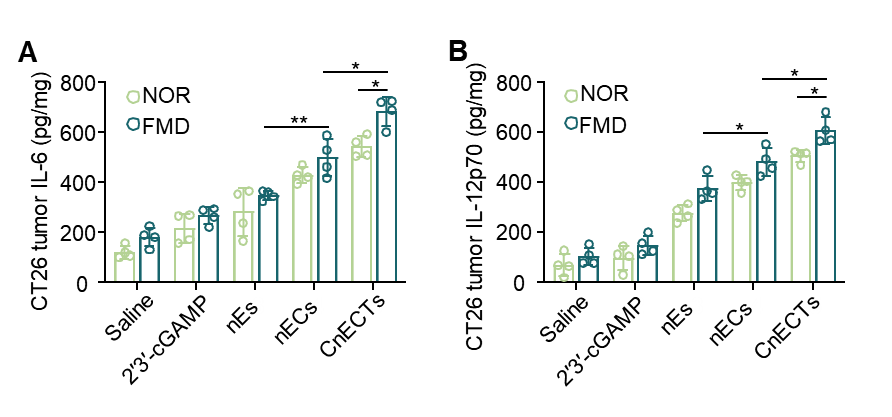


**Figure S38.** The levels of (A) IL-6 and (B) IL-12p70 in the CT26 tumors were measured by ELISA kits. Error bars represent ± s.d. (n = 4). Statistical significance was assessed by one-way ANOVA with turkey test. **p* < 0.05, ***p* < 0.01.


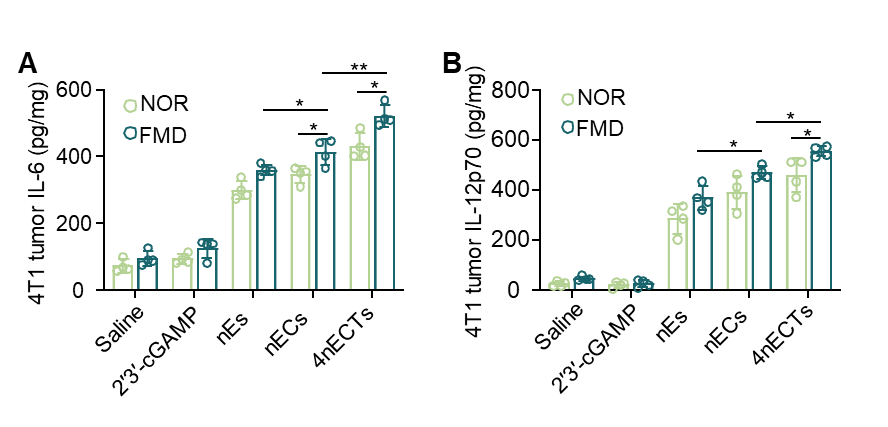


**Figure S39.** The levels of (A) IL-6 and (B) IL-12p70 in the 4T1 tumors were measured by ELISA kits. Error bars represent ± s.d. (n = 4). Statistical significance was assessed by one-way ANOVA with turkey test. **p* < 0.05, ***p* < 0.01.


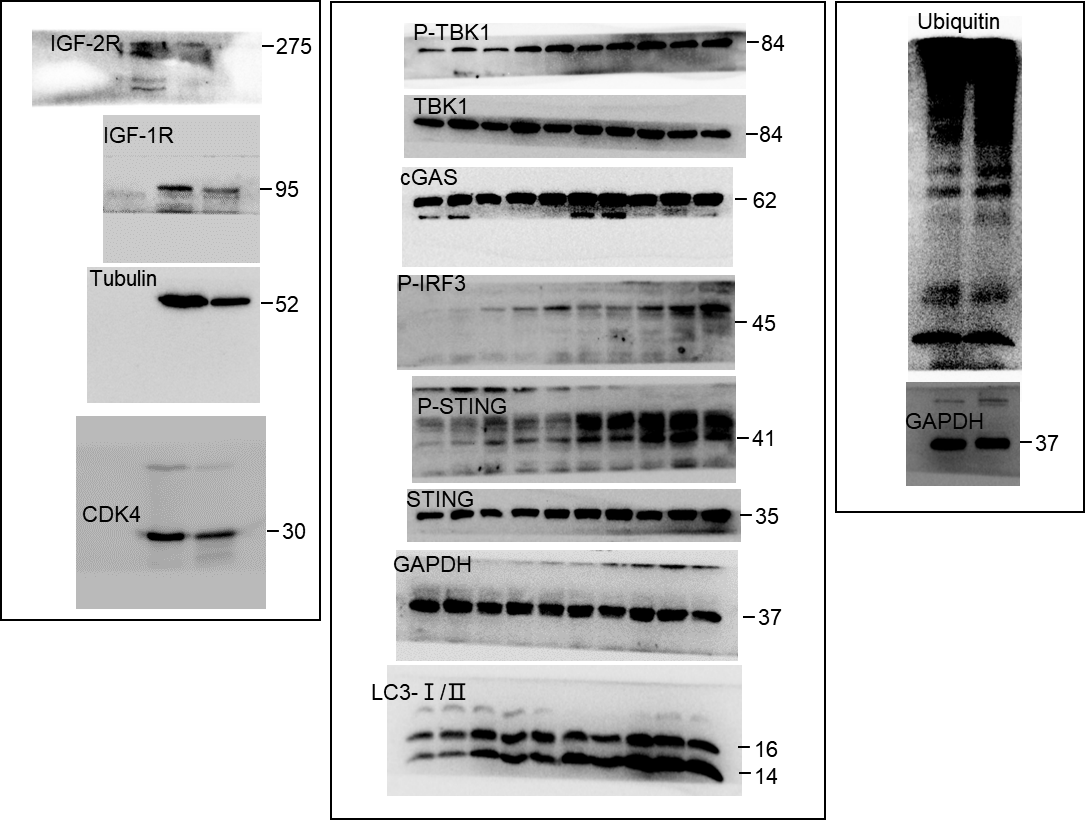


**Figure S40.** Unprocessed images of immunoblots.

**
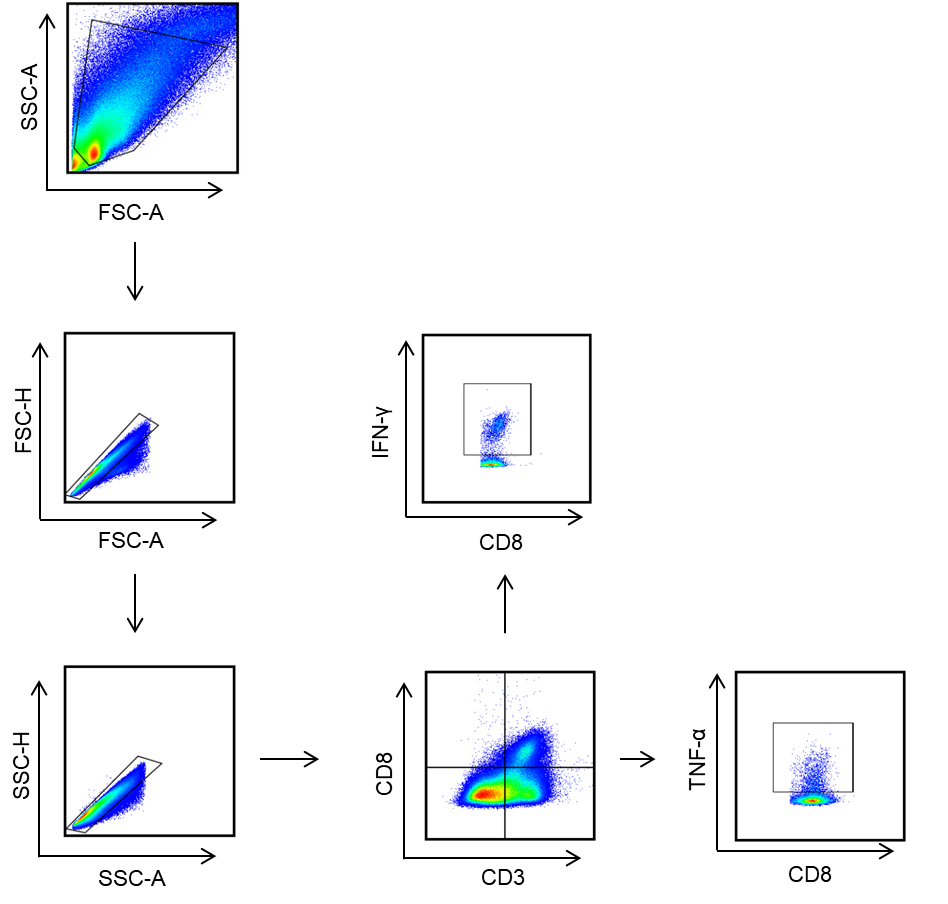
**

**Figure S41.** Flow cytometric gating strategy for isolating immune cell populations in the lymph nodes. Cells were stained separately with distinct panels of antibody-conjugated fluorophores for analysis.


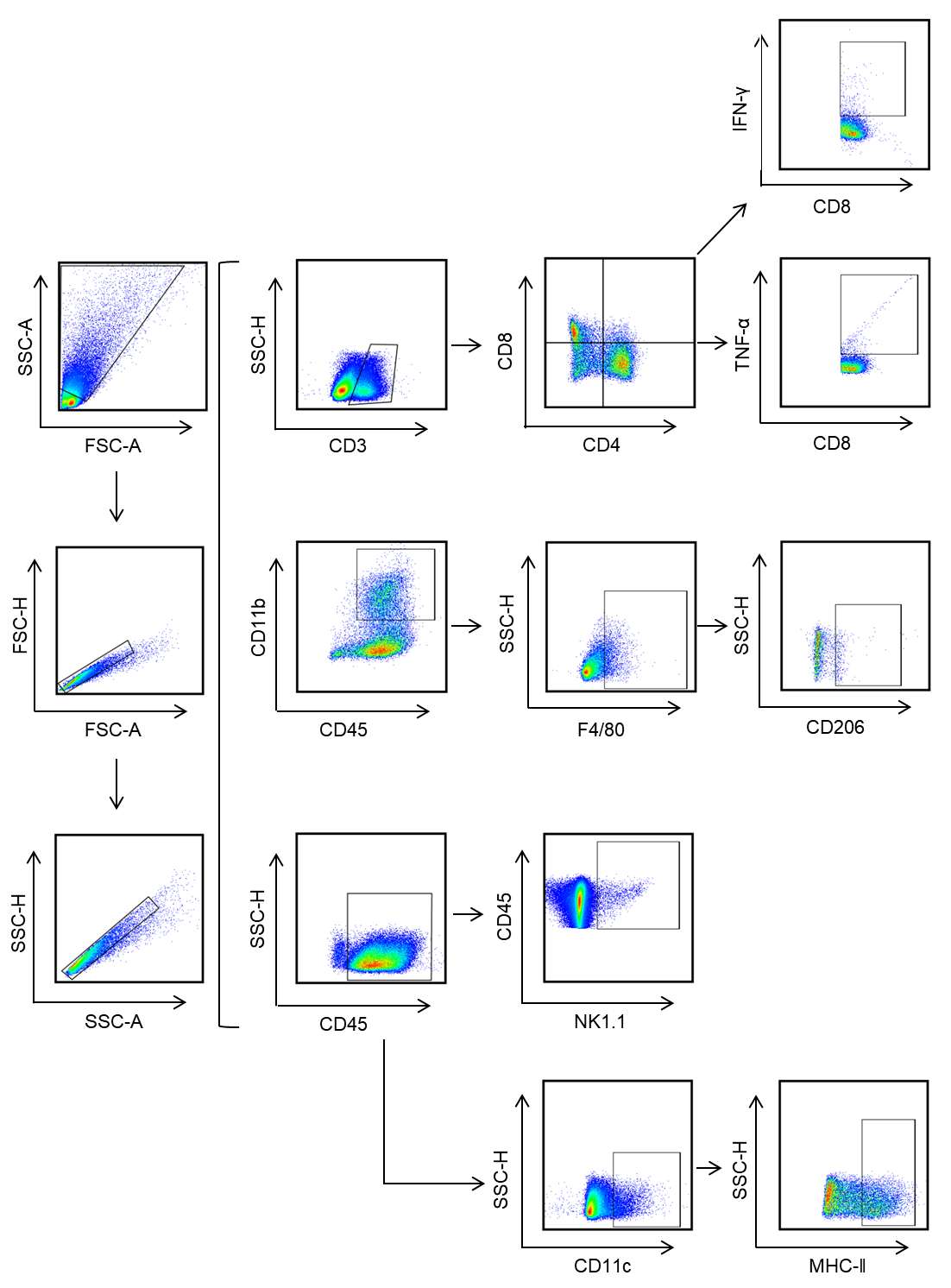


**Figure S42.** Flow cytometric gating strategy for isolating immune cell populations in the tumor immune microenvironment. Cells were stained separately with distinct panels of antibody-conjugated fluorophores for analysis.

**Table S1.** Abbreviations in the manuscript.

| **Abbreviation** | **Definition** |
| --- | --- |
| STING | Stimulator of interferon genes |
| 2'3'-cGAMP@*E.coli* | Genetically engineered bacteria producing 2'3'-cGAMP |
| nECTs | 2'3'-cGAMP@*E.coli* with autologous tumor antigens |
| TILs | Tumor-infiltrating lymphocytes |
| cGAS | Cyclic GMP-AMP synthetase |
| APCs | Antigen-presenting cells |
| DCs | Dendritic cells |
| m-cGAS | Mouse-cGAS |
| FMD | Fasting-mimicking diet |
| IPTG | Isopropyl β-D-1-thiogalactopyranoside |
| TEM | Transmission electron microscopy |
| nECs | Nano-sized E. coli protoplasts |
| DLS | Dynamic light scattering |
| BnECTs | 2'3'-cGAMP@*E.coli* with B16-F10 tumor antigens |
| OnECTs | 2'3'-cGAMP@*E.coli* with B16-OVA tumor antigens |
| CnECTs | 2'3'-cGAMP@*E.coli* with CT26 tumor antigens |
| 4nECTs | 2'3'-cGAMP@*E.coli* with 4T1 tumor antigens |
| IGF-1R | Receptors for insulin-like growth factor 1 |
| IGF-2R | Receptors for insulin-like growth factor 2 |
| CDK4 | Cyclin-dependent kinase 4 |
| LPS | Lipopolysaccharide |
| HPLC | High-performance liquid chromatography |
| p-IRF3 | Phosphorylated IRF3 |
| p-TBK1 | Phosphorylated TBK1 |
| p-STING | Phosphorylated STING |
| ER | Endoplasmic reticulum |
| BMDCs | Bone marrow-derived dendritic cells |
| IL-6 | Interleukin-6 |
| TNF-α | Tumor necrosis factor-α |
| NK | Natural killer |
| ALT | Alanine aminotransferase |
| ALP | Alkaline phosphatase |
| AST | Aspartate aminotransferase |
| CK | Creatine kinase |
| FITC | Fluorescein isothiocyanate |
| ICG | Indocyanine green |
| PMSF | Phenylmethylsulfonyl fluoride |
